# Origami of KR-12 Designed Antimicrobial Peptides and Their Potential Applications

**DOI:** 10.3390/antibiotics13090816

**Published:** 2024-08-28

**Authors:** Jayaram Lakshmaiah Narayana, Abraham Fikru Mechesso, Imran Ibni Gani Rather, D. Zarena, Jinghui Luo, Jingwei Xie, Guangshun Wang

**Affiliations:** 1 Department of Pathology, Microbiology, and Immunology, College of Medicine, University of Nebraska Medical Center, 985900 Nebraska Medical Center, Omaha, NE 68198, USA; dr.jayaramln@gmail.com (J.L.N.); amechesso@unmc.edu (A.F.M.); irather@unmc.edu (I.I.G.R.); zareenajntua@gmail.com (D.Z.); 2 Department of Biotechnology, Dayananda Sagar College of Engineering, Bangalore 560078, India; 3 College of Engineering, Jawaharlal Nehru Technological University, Anantapur 515002, India; 4 Department of Biology and Chemistry, Paul Scherrer Institute, 5232 Villigen, Switzerland; jinghui.luo@psi.ch; 5 Department of Surgery-Transplant and Mary & Dick Holland Regenerative Medicine Program, College of Medicine, University of Nebraska Medical Center, Omaha, NE 68198, USA; jingwei.xie@unmc.edu

**Keywords:** antimicrobial peptides, KR-12, LL-37, lipopeptides, macrocyclic peptides, stapled peptides, Trp-caged peptides

## Abstract

This review describes the discovery, structure, activity, engineered constructs, and applications of KR-12, the smallest antibacterial peptide of human cathelicidin LL-37, the production of which can be induced under sunlight or by vitamin D. It is a moonlighting peptide that shows both antimicrobial and immune-regulatory effects. Compared to LL-37, KR-12 is extremely appealing due to its small size, lack of toxicity, and narrow-spectrum antimicrobial activity. Consequently, various KR-12 peptides have been engineered to tune peptide activity and stability via amino acid substitution, end capping, hybridization, conjugation, sidechain stapling, and backbone macrocyclization. We also mention recently discovered peptides KR-8 and RIK-10 that are shorter than KR-12. Nano-formulation provides an avenue to targeted delivery, controlled release, and increased bioavailability. In addition, KR-12 has been covalently immobilized on biomaterials/medical implants to prevent biofilm formation. These constructs with enhanced potency and stability are demonstrated to eradicate drug-resistant pathogens, disrupt preformed biofilms, neutralize endotoxins, and regulate host immune responses. Also highlighted are the safety and efficacy of these peptides in various topical and systemic animal models. Finaly, we summarize the achievements and discuss future developments of KR-12 peptides as cosmetic preservatives, novel antibiotics, anti-inflammatory peptides, and microbiota-restoring agents.

## 1. Introduction

A 2019 study estimated that antibiotic-resistant bacterial infections cause 1.2 million deaths globally [1]. This number is projected to reach 10 million by 2050 [2]. Most of the hospital-associated infectious diseases are caused by the six highly virulent and drug-resistant ESKAPE pathogens, including *Enterococcus faecium*, *Staphylococcus aureus*, *Klebsiella pneumoniae*, *Acinetobacter baumannii*, *Pseudomonas aeruginosa*, and *Enterobacter* spp. The development of various resistance mechanisms of these pathogens (e.g., efflux pumps and drug inactivation) poses challenge to eliminate them, leading to the bad bugs no drugs problem [3]. Among them, Gram-negative pathogens are more problematic because of the difficult-to-permeate two layers of membranes and efficient drug efflux pumps [4]. In addition, these pathogens can be hidden in biofilms, which are non-permeable to small molecule drugs and immune cells. Some pathogens, such as methicillin-resistant *S. aureus* (MRSA), can also live within cells, thereby dodging the action of small antibiotics that cannot enter cells [5]. Hence, a new generation of antimicrobials is urgently needed to eliminate these pathogens in both planktonic and biofilm forms.

Antimicrobial peptides (AMPs) have been demonstrated to be effective against antibiotic-resistant pathogens, persisters, and their biofilms [6,7,8]. As cationic peptides, AMPs can kill pathogens rapidly by acting on anionic bacterial membranes [9,10], making it difficult for pathogens to develop resistance. According to the Antimicrobial Peptide Database (APD; https://aps.unmc.edu), at least 3261 natural AMPs with demonstrated activity have been discovered from a variety of organisms (accessed on 29 July 2024) [11,12]. The sources of these peptides have been classified into six life kingdoms, including bacteria, archaea, protists, fungi, plants, and animals. Among these peptides, approximately 150 originate from humans [13]. Although dozens of defensin genes have been mapped in the human genome [14], there is only one cathelicidin gene (CAMP) in *Homo sapiens* [15]. The *camp* encodes a precursor protein of 18 kDa (hCAP-18) where the cathelin domain is highly conserved, while the C-terminal antimicrobial region varies substantially in terms of amino acid sequence, 3D structure and activity spectrum. The mature peptide of hCAP-18 was initially predicted as FALL-39 [16]. This predicted peptide is two residues longer than the natural peptide LL-37 isolated from neutrophils [17] and one residue longer than ALL-38, a processed form of male sperm hCAP-18 after injection into vagina during sex intercourse [18]. Such a remarkable accuracy in predicting FALL-39 makes sequence alignment the most reliable method for identification of cathelicidins in sequenced genomes. By the time this manuscript was completed, 159 vertebrate cathelicidins have been reported to be antimicrobial and documented in the APD [11].

Elucidation of the host defense mechanisms of plants and animals can also provide new strategies to fight pathogenic infection. Insects are capable of expressing different AMPs in response to bacterial and fungal infections. There are different molecular pathways for recognition, expression, and secretion of insect AMPs. The Toll (pathogen receptor) signaling pathway recognizes Gram-positive bacteria and fungi and produces peptides to eliminate these pathogens via the NF-κB like transcription factor, whereas the IMD (immune deficient) pathway is designed to express AMPs to fight Gram-negative pathogens [19]. Subsequently, Toll-like receptors (TLRs) have also been found in vertebrates, including humans, as pathogen-recognition systems [20]. Different pathogen-associated molecular patterns can trigger different TLRs. Vitamin D receptor is linked with the TLR to induce human beta defensin (hBD) and cathelicidin LL-37 [21], which can work synergistically in killing bacteria [22]. Interestingly, human cathelicidin can be expressed under sunlight as well [23].

**Figure 1:**
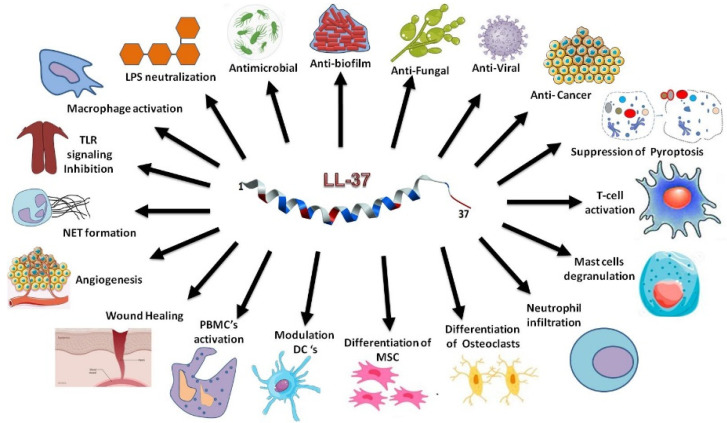
Properties and functions of human cathelicidin LL-37 discovered in different cells. Depicted in the center is the 3D structure of membrane-bound LL-37 (PDB ID: 2K6O) determined by 3D triple-resonance heteronuclear multidimensional nuclear magnetic resonance (NMR) spectroscopy. When targeting bacterial membranes, the C-terminal tail of LL-37 is not folded and remains highly flexible as confirmed by heteronuclear ^15^N backbone dynamics on the ps-ns time scale [32]. The C-terminal tail is disordered in complex with SDS, D8PG, and LPS (abbreviations in the text). Direct interactions of LL-37 with anionic bacterial phosphatidylglycerols (PGs) and LPS as demonstrated by NMR provide basis for antimicrobial and anti-inflammatory effects. NET: neutrophil extracellular traps; PBMC: peripheral blood mononuclear cells; DCs: dendritic cells; MSC: mesenchymal stem cell.

LL-37, detected in various human epithelial cells and organs (Figure 1), can protect the host from infection [24,25,26]. Significantly, this host defense peptide is able to inhibit bacterial biofilm formation as well [27]. It also neutralizes TLR-4 ligand lipopolysaccharides (LPS) and displays anti-inflammatory activity by suppressing cytokines such as IL-1β and IL-6. LL-37 regulates the activity of immune cells such as macrophages, dendritic cells, and T cells in different manners (Figure 1). It stimulates the production of type I interferon and activates immune cells to boost adaptive immunity as well [28,29,30]. Our picture on host defense continues to expand. It has been appreciated that commensal bacteria also play an indispensable role in shaping our host defense. These “good bacteria” can secrete bacteriocins (i.e., bacterial AMPs) to fend off “bad bacteria”. Interestingly, human LL-37 can work synergistically with bacteriocins to protect skin from infection, offering yet another strategy to control invading pathogens [31].

**Figure 2:**
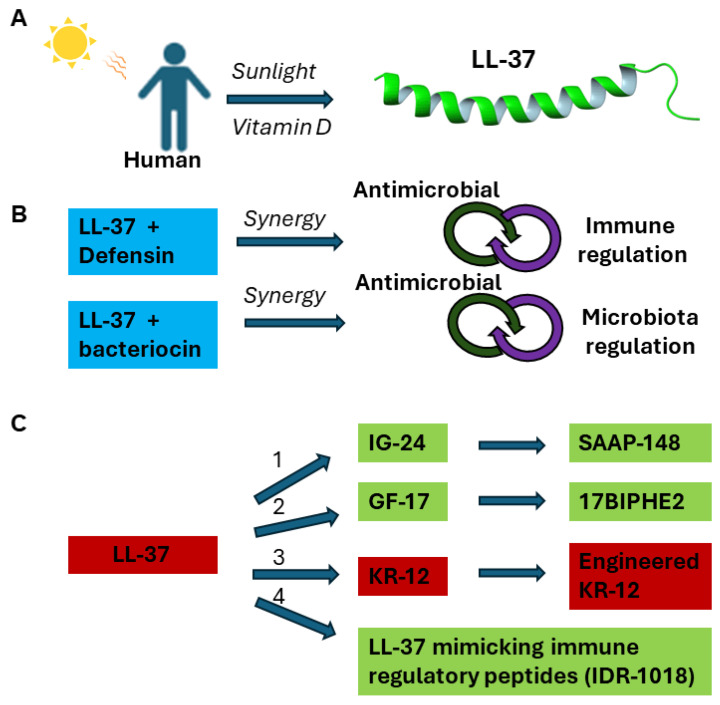
Therapeutic strategies based on human cathelicidin LL-37. (**A**) Humans can use sunlight or vitamin D and its analog to switch on the expression of LL-37 to boost innate defense against infection. Likewise, recombinant DNA technology can be used to express LL-37 to achieve the same production. (**B**) Human LL-37 can function synergistically with other human AMPs such as defensin or lysozyme to better control pathogens. Similarly, human LL-37 can work synergistically with bacteriocins from commensal bacteria to better control invading pathogens. Using the same strategy, AMPs can be used with existing antibiotics to overcome resistance. (**C**) LL-37 can be engineered into novel antimicrobial agents based on different fragments (IG-24, GF-17, and KR-12) discovered from (1) peptide library, (2) structure-based design, (3) combined (1) and (2), and (4) feature-based mimicking (reviewed in ref. [26]). This review focuses on a variety of the engineered constructs based on KR-12, the smallest antibacterial fragment of LL-37.

The multiple functions of LL-37 laid a foundation for its potential applications. Figure 2 summarizes major strategies for developing LL-37 based therapeutics. Vitamin D can induce the expression of human LL-37 [33], which can target the anionic membranes of pathogens via forming a long amphipathic helix with the C-terminus unstructured [32]. Vitamin D and other LL-37 inducing agents, such as phenylbutyrate [13], have been subjected to clinical trials as a potential treatment strategy [34] (Figure 2A). LL-37 cream has been shown to enhance the healing rate of diabetic foot ulcers (DFUs) with mild infection [35]. In its natural state, LL-37 can function synergistically with other host AMPs [22] or even commensal bacteriocin [31]. Likewise, a better effect may be achieved by combining AMPs with conventional antibiotics (Figure 2B). LL-37 fragments have been engineered for antimicrobial and spermicidal uses (Figure 2C) [36]. Short peptides (e.g., IDR-1018) have been developed to mimic the immune regulatory role of LL-37 [37]. In addition, LL-37 shows potential as an anticancer therapy by inducing apoptosis in certain types of cancer cells. Shorter peptides of LL-37 (e.g., FK-16) may have superior anticancer and antimicrobial properties [26,38,39,40,41,42].

Since our last review on the progress made with the design of LL-37 in 2019 [26], exciting constructs have been made, primarily based on KR-12 since its discovery in 2008 [32]. Table 1 provides the timeline for select constructs of KR-12, the smallest selective antimicrobial peptide derived from human LL-37 [32]. This review focuses on the discovery, design, and applications of KR-12. First, we summarize the discovery, 3D structure, and structure–activity relationship of KR-12 itself. Then, we describe a variety of peptide constructs designed based on the KR-12 template. Subsequently, we review toxicity and in vivo efficacy studies of KR-12-derived peptides in different animal models. These results pave the way for potential applications of the engineered constructs of KR-12.

## 2. Discovery, NMR Structure, and Mechanism of Action of KR-12 Derived from the Antimicrobial Core of Human LL-37

### 2.1. The Discovery of KR-12

**Figure 3:**
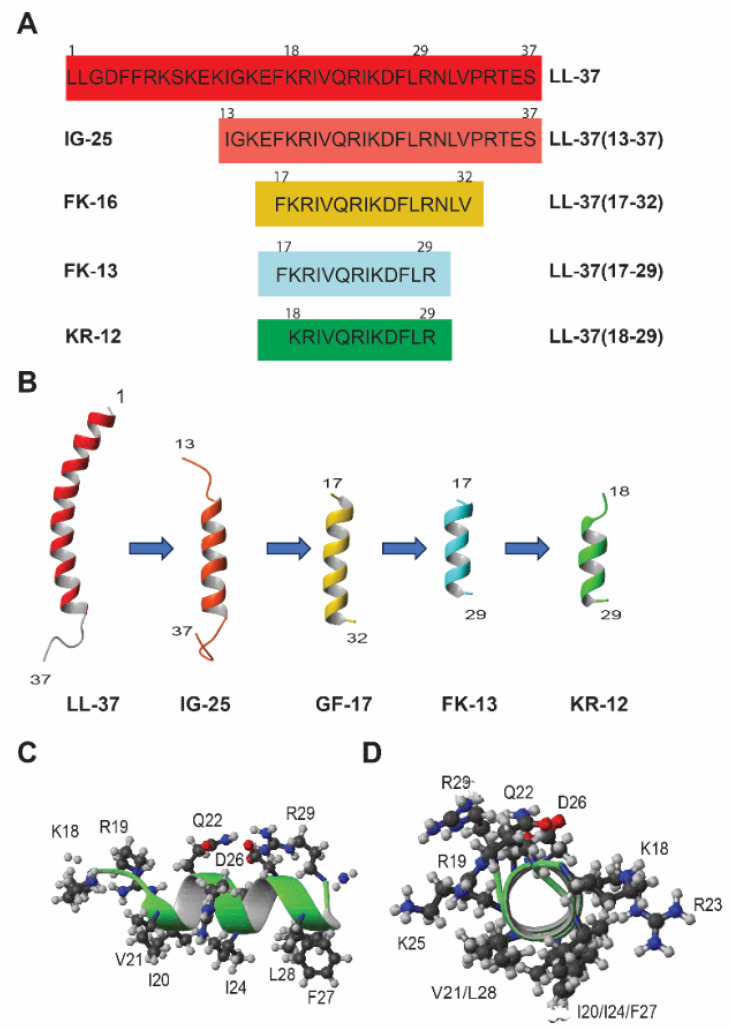
The discovery path of KR-12 through structural studies. (**A**) Amino acid sequences and nomenclature of LL-37 and its fragments. The original peptide names for these fragments are given on the right of the peptide sequences, while the shortened names are provided on the left. While LL-37 has a carboxylic acid at the C-terminus, the C-termini of shorter sequences, including FK-16/GF-17, FK-13, and KR-12, are all amidated to increase the net charge by +1. (**B**) Backbone structures of LL-37, IG-25, GF-17, FK-13, and KR-12 determined by 2D and 3D NMR spectroscopy [32,39,60]. Except for LL-37 and IG-25, GF-17, FK-13, and KR-12 are C-terminally amidated. (**C**,**D**) Horizontal and vertical views of the NMR structure of KR-12 in complex with anionic D8PG [32].

Human LL-37 has 37 residues and is too costly to synthesize chemically as a new antibiotic. In addition, LL-37 can be rapidly degraded by proteases and lose activity in serum [42]. Hence, there is a strong desire to identify its active regions followed by peptide engineering (Figure 2C). By using peptide libraries [56,57,58,59] and through structural studies [39], numerous LL-37 fragments have been identified (Figure 2). The major fragments have been highlighted in previous review articles [24,25,26]. These fragments have improved our understanding of the sequence-activity relationship of human LL-37. Wang discovered the smallest antibacterial peptide KR-12 through a combination of structural and library approaches, starting with the first study of human LL-37 by two-dimensional (2D) NMR spectroscopy [32,39]. Although LL-37 comprises only 37 amino acids, it still poses challenges for structural determination by 2D NMR spectroscopy because of line broadening and spectral overlap after micelle binding. Hence, a cut-and-conquer strategy was adopted by splitting LL-37 into two fragments: LL-37(1-12) (also called LL-12) and LL-37(13-37) (also known as IG-25). The fragment IG-24 (i.e., P60.4Ac or OP-145 obtained from library screen) [56] for designing SAAP-148 [59] does not have the C-terminal residue of IG-25. For convenience, peptide amino acid sequences, including both the original names (e.g., LL-37(18-29)) and the shortened names (e.g., KR-12), are given in Figure 3A. The structure of LL-37(13-37) suggested that both terminal residues could be removed since they are either poorly defined or disordered structures (Figure 3B). Correspondingly, those amino acids in the structured region gave weak signals due to membrane binding (leading to short relaxing time T_2_), while those in the randomly coiled region yielded strong NMR signals. This NMR-trim technology (originally called TOCSY-trim) allowed us to trim residues with strong signals and identify the major antimicrobial peptide FK-16 [39]. GF-17, which contains an extra glycine the N-terminus of FK-16, is indeed all helical (Figure 3B) [60]. The addition of this glycine had little effect on peptide activity against a panel of Gram-positive and Gram-negative bacterial pathogens [26]. In addition, FK-16 and GF-17 are useful candidates for anticancer and antiviral studies [40,61]. Due to weak binding to micelles, residues 30-32 were deleted from FK-16 as well, leading to the identification of LL-37(17-29), the core antimicrobial peptide of LL-37. This core peptide (also called FK-13) is entirely helical in complex with membranes (Figure 3B) [39]. The FK-13 peptide remains antibacterial and helical after sequence reversal (retro-FK13) [62]. From a pool of shorter peptides, KR-12 (residues 18–29 of LL-37) was identified as the smallest antibacterial peptide against *Escherichia coli* K12 but not *S. aureus* USA300 [32]. Note that KR-12 is the smallest antibacterial peptide, whereas FK-13 is the minimal anti-human immunodeficiency virus (HIV) peptide (For a review, see ref. [26]). In 2018, Jenssen and colleagues confirmed KR-12 as a small antimicrobial, antibiofilm, and immune-modulating peptide based on a library screening of overlapping 12-residue LL-37 peptides [63]. In addition, they found FK-12, which does not have the last arginine of the LL-37 core peptide FK-13, inhibited the growth and biofilm formation of *Staphylococcus epidermidis*.

### 2.2. Structural Determination and Mechanistic Studies of KR-12

A peptide can be in different states: lyophilized powder form, crystal, in solution, and bound to membranes. Fourier-transform infrared spectroscopy (FT-IR) studies provide evidence that KR-12 may be in different conformations [64]. This review only discusses the active structures of KR-12 when bound to bacterial membranes. KR-12 is found to act on membranes [64] in a similar manner to its parent peptide LL-37 via carpet model [65]. In such a state, the amphipathic structure is a key to understanding the antimicrobial action of cationic peptides. Both basic and hydrophobic amino acids play a critical role in targeting anionic bacterial membranes. Database statistics reveals that basic amino acids are essential for killing Gram-negative bacteria. In contrast, hydrophobic amino acids are more important to kill Gram-positive pathogens such as MRSA [11,12]. While basic arginine (R) and lysine (K) can initiate bacterial recognition via long-range electrostatic interactions, hydrophobic amino acids can anchor the peptide to bacterial membranes via Van der Waals forces. It appears that arginine with a bifurcated sidechain is more powerful than lysine, and replacement of lysine with arginine frequently increases peptide activity [66,67,68]. In many cases, the evidence for membrane action is indirect (e.g., via the fluorescence of a dye). It is rare to report direct interactions between peptide and bacterial membranes. NMR is a technology that enables not only structural determination but also observation of peptide–lipid interactions. In addition, NMR provides insight into protein/peptide dynamics [69,70].

Because of the complexity of biological membranes, structural studies by solution NMR are normally carried out in membrane-mimetic systems. Two commonly used membrane-mimetic models are anionic sodium dodecyl sulfate (SDS) and zwitterionic dodecylphosphocholine (DPC) micelles [71,72]. The small sizes of these micelles enable them to tumble rapidly in solution, yielding sharp spectral lines. In addition, deuteration of SDS and DPC can get rid of interference of protons from these micelles. However, neither SDS nor DPC represents the head group of bacterial anionic lipids phosphatidylglycerols (PGs) or LPS. The Wang lab explored the use of dioctanoylphosphatidylglycerol (D8PG) for structural studies of AMPs [32,73,74]. D8PG, which shares the same head group with PGs, provides a better mimic of bacterial membranes than SDS. Moreover, NMR measurements suggest that D8PG form larger lipid micelles than SDS [32].

KR-12 starts with amino acids K and R and consists of 12 amino acids with a net charge of +5 due to C-terminal amidation (Table 1). For consistency and to facilitate a correlation between the fragments and intact peptide, the LL-37 numbering was retained for KR-12 (residues K18 to R29) in our published papers (Figure 3). KR-12 indeed utilizes an amphipathic helix structure to associate with D8PG. The helical structure can be clearly seen in the backbone view of KR-12 in Figure 3B, while the amphipathic nature of KR-12 can be viewed in Figure 3C,D. Since D8PG is protonated, it also allowed us to observe peptide-PG interactions (Arg-PG and aromatic Phe-PG) directly by intermolecular NOESY spectroscopy [32]. These nuclear Overhauser effect (NOE) contacts provide solid evidence for electrostatic and hydrophobic interactions between AMPs and D8PG [32,74]. In particular, only interfacial R23 can directly interact with PGs. It was hypothesized that the positive charges of KR-12 (Figure 3C) could attract bacterial anionic lipids [32]. This lipid clustering mechanism of KR-12 was substantiated in our collaborative studies with Richard Epand [75]. KR-12 also permeates bacterial inner membranes (see below). In addition, a multiple-turn amphipathic structure was observed for KR-12 in the presence of *E. coli* LPS based on transferred NOESY spectroscopy (Figure S1). In the case of LL-37, the long helical structure in complex with SDS or D8PG (Figure 3B) was determined by 3D NMR spectroscopy for high resolution. When in complex with *E. coli* LPS, the C-terminal tail of LL-37 remains disordered and observable, while the rest peptide signals broadened into the baseline due to LPS binding. In addition, ^15^N backbone dynamics delineates the rigid and mobile regions, validating the micelle-bound 3D structure of LL-37 determined independently by distance and dihedral angle restraints [32]. Hence, LL-37 and KR-12 provide two examples where NMR studies have been conducted in both D8PG and LPS, providing direct evidence for membrane targeting and structural basis for LPS-mediated anti-inflammation [32].

**Figure 4:**
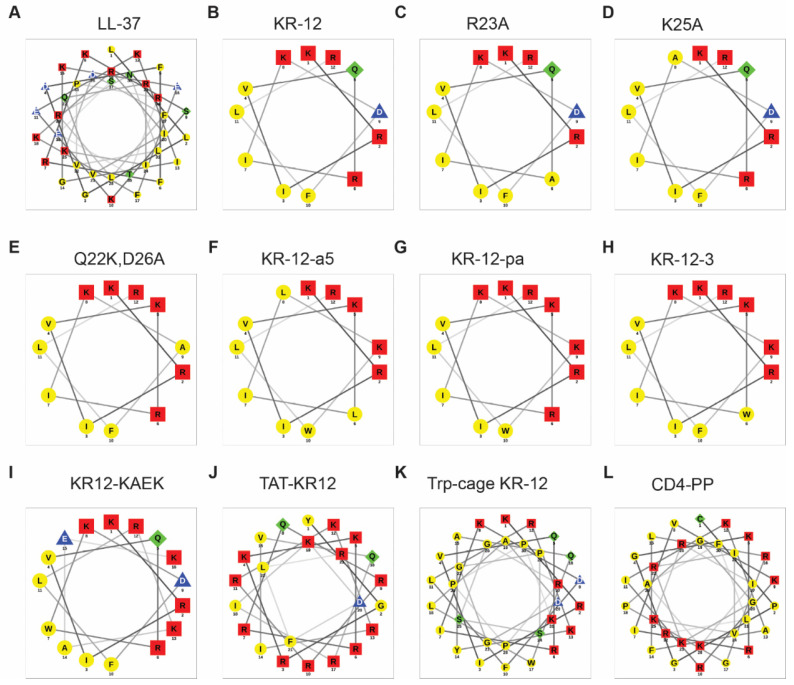
Helical wheel plots for LL-37 (**A**), KR-12 (**B**) and its selective derivatives (**C**–**L**). The helical wheel was generated using the NetWheel program (http://lbqp.unb.br/NetWheels/, accessed on 31 July 2024). In this program, amino acids are classified into four groups: (1) polar/basic (red square): RHK, (2) polar/acidic (blue triangle): DE, (3) polar/uncharged (green diamond): STNQC, and (4) nonpolar (yellow circle): AGVILMFYWP. Although included in the plot, it is evident that the HIV TAT sequence is not amphipathic (**J**). However, the amphipathic helical structure can still be seen in the presence of additional sequence from the Trp cage (**K**). Finally, some symmetry can be seen in the helical wheel plot of cyclic KR-12 dimer (**L**).

Since the helical wheel plot [76] is widely utilized, we compared the helical wheel plots of LL-37 (Figure 3A) and KR-12 (Figure 3B) with their NMR structures (Figure 3B,D). The hydrophobic moment of LL-37 (0.521) is lower than that of KR-12 (0.782). However, they have very similar hydrophobicity values (LL-37: 0.201 vs. KR-12: 0.193). Both LL-37 and KR-12 form clear amphipathic helices with hydrophilic and hydrophobic side chains well separated in two sectors. However, neither LL-37 nor KR-12 is entirely helical (Figure 3) based on the NMR study [32]. While the C-terminal tail of LL-37 is disordered in complex with PGs and LPS, the N-terminal K18 and R19 are less well defined in the NMR structure of KR-12 bound to D8PG. Moreover, the positions of the KR-12 side chains in the helical wheel (Figure 4B) are evenly distributed. However, they are aligned in the NMR structure due to hydrophobic packing (Figure 3D). Because not every KR-12 construct has a determined 3D structure, we plotted the helical wheels for select KR-12 variants to facilitate our discussion (Figure 4).

## 3. Structure–Activity Relationship (SAR) of KR-12

### 3.1. Molecular Forms and Media Conditions Influence Peptide Activity

One of the challenges in preparing this review was the comparison of KR-12 activity results reported by different laboratories. The activity differences for KR-12 could result from peptide forms, media conditions, bacterial strains, and detection methods. KR-12 have been made in different forms: acetylated, amidated, or both. The original KR-12 peptide synthesized in the Wang laboratory contains C-terminal amidation without N-terminal acetylation (Table 1). In Mueller Hinton Broth (MHB), this KR-12 inhibited *E. coli* with a minimum inhibitory concentration (MIC) at 40–66 µM but did not inhibit MRSA even at a very high concentration (>264 µM) [32,75]. When the C-terminus was not amidated, the peptide lost its activity (Asaf Sol and Gilad Bachrach, personal communication). With N-terminal acetylation, KR-12 showed activity against *S. aureus* but not *E. coli* [55]. Such an activity spectrum is opposite to that of the original KR-12 without acetylation [75]. Hence, the activity of KR-12 containing a minimal sequence for activity is highly sensitive to peptide end capping.

Environmental conditions are known to influence peptide activity. Histidine-rich peptides work best at acidic condition when it is protonated and cationic [77], while daptomycin and SAAP peptides, containing a string of aspartic acids, are only active in the presence of metal [78,79]. Culturing media may also influence peptide activity. While Zhang et al. reported that LL-37 was inactive against a panel of Gram-positive and -negative bacteria [55], Jacob and colleagues found that LL-37 was able to kill a panel of bacteria in a sensitive medium [80]. In addition, Luo et al. showed that LL-37 and KR-12 inhibited both *E. coli* and *S. aureus* [81]. However, when tested in the recommended standard MHB, LL-37 was lethal to *E. coli* but did not kill *S. aureus* USA300 [82,83]. Lehrer and colleagues revealed the effects of anionic components in media on LL-37 activity since LL-37 became active after removal of these anionic components from media [82]. We observed LL-37 activity against MRSA when the MHB was diluted to ~10% [83]. In the diluted media, KR-12 and LL-37 are equally active against both *E. coli* and *S. aureus*. These results underscore the importance of both the molecular forms of KR-12 and media conditions for antimicrobial activity. We recommend the use of sensitive conditions during antimicrobial screening to increase the positive hits. However, adherence to the standard antimicrobial protocols during structure–activity relationship studies and when finally reporting peptide activity can ensure comparability of the MIC results from different laboratories. The use of standard American Type Culture Collection (ATCC) strains, plate type, and the inclusion of known antibiotics are also useful for data comparison. For additional considerations, including the role of cation (e.g., Mg^2+^ and Ca^2+^), readers are strongly suggested to refer to the helpful protocol for aerobic bacteria from the Hancock lab [84]. Although µg/mL is widely used, we also recommend the use of ultraviolet (UV) spectroscopy to quantify fresh peptides and represent MIC values in µM [39], especially when comparing peptides with different lengths.

### 3.2. Alanine Scan

Alanine scanning is a powerful technique for understanding protein structure, function, stability, and interactions with other proteins or ligands. Mishra et al. reported the first study of alanine scan of cationic amino acids in KR-12, producing evidence for their distinct roles in peptide activity [43]. Membrane permeation experiments indicate that the R23A (hydrophobicity 0.303), K25A (0.301), and KR-12R (all lysine-to-arginine, 0.189) variants are more potent than KR-12 (0.193) against *E. coli*, while the double K18R19 variant and KR-12K (all arginine to lysine) are least effective. One of the convenient methods for SAR study of AMPs is circular dichroism (CD), which can follow conformational changes under different conditions. The majority of linear AMPs do not have a defined structure in aqueous solution. However, they become helical in membrane-mimetic environments. While most of the KR-12 variants are disordered, it is surprising that both the R23A (Figure 4C) and K25A (Figure 4D) variants display a helical feature even in phosphate-buffered saline (PBS). One possible reason is that the insertion of a hydrophobic alanine in the interface facilitates the formation of peptide oligomers via peptide-peptide interactions [43]. Another three cationic residues (K18, R19, and R29) of KR-12 (Figure 3D) on the hydrophilic face are more important in clustering anionic lipids and for hemolysis than R23 and K25 in the interfacial region. Thus, the location of basic amino acids in the amphipathic helix (Figure 3D) determines the role of an alanine in regulating peptide activity, membrane permeation, and oligomerization tendency. Recently, Luo and colleagues detected dimer, trimer, and tetramer of KR-12 at 0.5 µM according to an α-hemolysin nanopore and mass spectrometry study [85]. These oligomers are likely to account for a small population of KR-12 in solution. They may rise rapidly at high concentrations of peptide.

The LL-37 core peptide, LL-37(17-29) (i.e., FK-13), which contains only one extra N-terminal F17 compared to KR-12, forms a supramolecular structure in the crystal [86]. In this structure, acidic residue D26 forms both intermolecular and intramolecular salt bridges with adjacent arginines (R23 and R29). In addition, the aromatic–aromatic interaction between F27 in different molecules is critical for molecular packing into the basic four-helix bundle structure. Interestingly, such a helical fibril structure of LL-37(17-29), termed α-amyloid [86], differs from the β-sheet structure in β-amyloids [87]. Alanine mutational studies of this LL-37 core peptide reveals the important role of I24 or F27 in inhibiting the growth of *Micrococcus luteus* (MIC > 100 µM). Compared to the parent peptide FK-13 (MIC 22 µM), peptide activity of both F17A and K18A variants went down (MIC 60 µM), while the Q22A substitution only had a minor effect on the activity (MIC 33 µM) [86]. The formation of oligomers is proposed here to play a role in bacterial killing of the peptide. This is also proposed for AMPs with β-sheet structures [88]. Future studies may depict a more complete picture for the relationship between peptide oligomerization and membrane mediated bacterial killing by helical LL-37 and its fragments.

LL-37 and its long fragments can also form oligomers [89,90,91], and aromatic–aromatic interactions involving F5/F6 probably play a role [92]. A classic picture is that LL-37 oligomers are concentration dependent and can disintegrate into monomers upon binding bacterial membranes and exert their effects in a bacteria type and peptide concentration-dependent manner [65]. This peptide oligomerization picture with and without membranes may evolve with the accumulation of new data, which may facilitate structural prediction of peptide aggregation and fibril formation via AlphaFold [93] or advanced AI tools under development.

Gunasekera et al. conducted a complete alanine scan of KR-12 [94] using a sensitive condition for activity evaluation by first incubation in a buffer followed by incubation in the media (peptide sequences in Table S1). This study shines light on the role of each amino acid of KR-12 in inhibiting the growth of bacteria. They identified that Gln5 (Q22 in LL-37) and Asp9 (D26) are key positions to modulate antibacterial activity (helical wheel in Figure 4E). The Q22K/D26A variant displayed up to an eight-fold improvement in antimicrobial activity compared to KR-12. This KR-12 analog, like KR-12, is much less hemolytic than LL-37, laying the foundation for its use in synthesizing macrocyclic dimeric KR-12.

## 4. Innovative Engineering Strategies of KR-12

**Figure 5:**
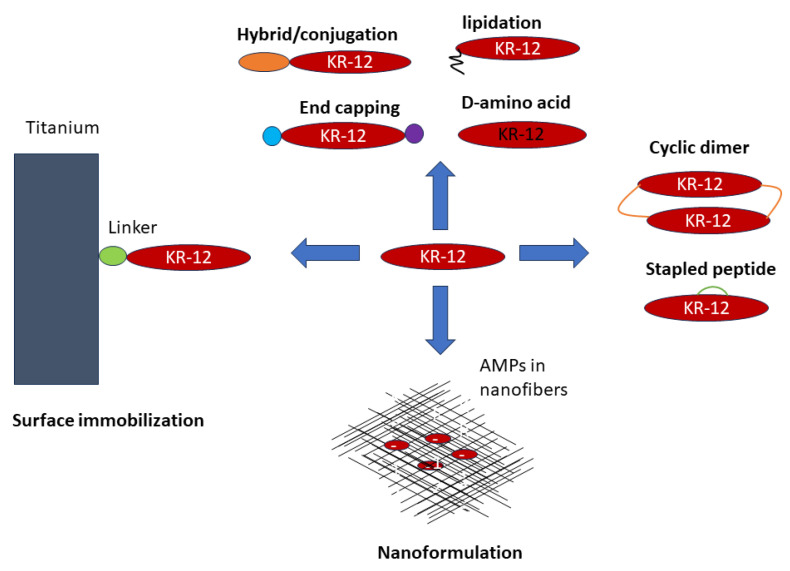
Various design strategies that transform human LL-37-derived KR-12 to new constructs: (1) amino acid changes, terminal capping, peptide hybridization, and peptide conjugates (**top**), (2) sidechain stapling and backbone cyclization (**right**), (3) surface immobilization (**left**), and (4) peptide formulation (**bottom**). See the text for further details. These strategies can be applied to other linear antimicrobial peptides as well.

Based on the connecting patterns of polypeptide chains, AMPs have been unified into four big classes: linear (UCLL), sidechain-linked (UCSS), sidechain-backbone-linked (UCSB), and backbone-linked peptides (UCBB) [95]. This section organizes various KR-12 constructs from linear to cyclic peptides based on this universal peptide classification system [96]. The primary design strategies applied to KR-12 include simple amino acid substitutions to enhance activity and reduce toxicity, terminal capping, incorporation of non-standard amino acids, stapled peptides, and peptide cyclization to enhance activity and stability to proteases. In addition, other strategies such as surface immobilization and nano-formulation have also been applied to KR-12. Figure 5 illustrates the state-of-the-art engineering of KR-12 into a variety of constructs (i.e., origami). Our discussion attempts to provide structural insight into the designed constructs whenever applicable.

### 4.1. Linear Analogs

#### 4.1.1. Amino Acid Substitutions

In 2013, Jacob et al. designed and synthesized a series of KR-12 analogs named KR-12-a1 to KR-12-a8 (peptide sequences in Table S1) [80]. The resulting peptides have both antimicrobial and antiendotoxic (anti-LPS) activities without mammalian cell toxicity except for KR-12-a6. They used a sensitive media condition with 1% peptone, which resulted in superior activity for all the peptides, including KR-12 and LL-37. Although the use of this medium might have obscured the activity changes due to amino acid substitutions in standard MHB, some subtle differences still persist. In particular, the substitution of R23 and K25 with a leucine reduced antibacterial activity of KR-12-a5 against *E. coli* and *P. aeruginosa* by two-fold and *S. typhimurium* by four-fold. These results support our earlier discovery that interfacial R23 and K25 are critical for killing Gram-negative pathogens [43,60]. This finding is also echoed in the hemolytic data, especially after replacement of both R23 and K25 with leucine. While most of the analogs are poorly hemolytic (HC_50_ > 800 µM), only KR-12-a5 (HC_50_ 96 µM) and KR-12-a6 (HC_50_ 22 µM) are much more hemolytic due to a substantial increase in peptide hydrophobicity by three-fold for KR-12-a5 and 4.25-fold for KR-12-a6. These two peptides show high affinity to LPS, thereby suppressing endotoxin-stimulated tumor necrosis factor-α (TNF-α) expression. Therefore, these KR-12 analogs have the potential for developing a new class of antimicrobial and anti-inflammatory therapeutic agents [80,97].

To mitigate the toxicity of KR-12-a5 (Figure 4F), Kim et al. have designed and synthesized a series of single-site D-amino acid-substituted analogs [47]. D-amino acids have a different configuration relative to L-amino acids more frequently observed in nature. Partial substitution with D-amino acids can alter peptide conformation. Wang proposes the concept of non-coherent packing to explain reduced peptide hydrophobicity and toxicity [39]. In the case of KR-12-a5, incorporating D-amino acids at positions 5, 6, or 7 significantly increased therapeutic index by 3.8- to 13.9-fold, while preserving its anti-inflammatory activity. Only the change at position 7 distorted peptide helical structure in the presence of 50% trifluoroethanol (TFE), SDS, or LPS. KR-12-a5 and its analogs showed a more potent antimicrobial activity than LL-37 and melittin against antibiotic-resistant bacteria, including clinically isolated MRSA, multi-drug-resistant *P. aeruginosa* (MDRPA), and vancomycin-resistant *Enterococcus* (VRE). Furthermore, compared to LL-37, KR-12-a5 and its analogs showed greater synergistic effects on MDRPA with conventional antibiotics, such as chloramphenicol, ciprofloxacin, and oxacillin (FICI between 0.25 and 0.5 for KR-12 peptides, but between 0.75 and 1.5 for LL-37). In addition, KR-12-a5 and its analogs were more effective antibiofilm agents against MDRPA than LL-37.

Yun et al. developed a natural antiseptic for cosmetics to replace parabens due to concerns with potential neurotoxic and allergic side effects [44]. They designed KR-12-pa (Table 1) and the formulated cosmetic displayed strong antimicrobial activity against various bacteria and fungi due to increased cationicity. The three substitutions (Q22K, D26K, and F27W) did not alter the amphipathic helical structure of KR-12 (Figure 4G), as confirmed by 2D NMR. Therefore, KR-12-pa may be included as a preservative in natural cosmetics.

Zhuo et al. produced a KR-12 variant named KR-12-3 (Table 1) by replacing three amino acids in KR-12: Q22K, R23W, and D26K [98]. With low cytotoxicity, KR-12-3 (helical wheel in Figure 4H) gained growth inhibitory activity against *Streptococcus gordonii* in planktonic and biofilm states. Further, both KR-12 and KR-12-3 could suppress LPS-induced IL-6 expression in murine RAW264 macrophages, indicative of anti-inflammatory capability.

#### 4.1.2. Peptide Hybrids and Conjugates with Enhanced Function

Combination of peptide motifs is a classic and effective approach to creation of new peptides with improved properties, spanning from enhanced activity to new functional capability. To enhance the activity of KR-12, da Silva et al. appended the KAEK segment to the C-terminus of the peptide [99]. These amino acids fit well into the amphipathic pattern in the helical wheel (Figure 4I). A search of the AMP database [12] reveals that this KAEK sequence is rare in natural AMPs and only present in AdCath, a cathelicidin from giant salamanders [100]. In addition, isoleucine at position I7 was changed to tryptophan. Since the resulting peptide [W7]KR12-KAEK gained substantial biofilm disruptive activity against *Enterococcus faecalis* strains, it is a promising antimicrobial agent to treat endodontic infections. Ajish and colleagues made a hybrid peptide by combining KR-12-a4 with LfcinB6 (a fragment derived from lactoferrin) to increase antimicrobial, anti-inflammatory, and antibiofilm activities [101]. To eliminate intracellular pathogens such as *S. aureus,* Huo et al. conjugated KR-12 with a cell-penetrating TAT motif, which smuggled KR-12 inside macrophages [48]. This TAT-KR-12 conjugate (Table 1 and Figure 4J) is more effective in eliminating Gram-positive bacteria both in vitro and in vivo. The conjugation of KR-12 with a carbohydrate-binding peptide (CBP) enabled its binding to bacterial-derived cellulose, a promising material for biomedical applications, including wound healing [54]. Of outstanding interest is that KR-12 has been fused with a Trp-cage domain to enhance peptide stability (Table 1). In this design, KR-12 is linked with a sequence KYAQWLADGGPSSGRPPPK, which can fold back around the Trp center to stabilize the cage structure through interactions with arginine and proline (P) [50]. This folded construct can stabilize portion of the KR-12 helical structure. This KR-12 construct gains stability to proteases, making it more suitable for practical application. It demonstrates increased bactericidal activity in physiological NaCl without sacrificing high cell selectivity of KR-12 [88]. As chemical synthesis of this sequence elongated KR-12 construct is costly, one may take advantage of conventional fermentation technology to produce the Trp-cage KR-12 at a large scale.

#### 4.1.3. Lipopeptides: Conjugates with Organic Acids

The properties of KR-12 can also be enhanced by conjugating with other chemical moieties. An organic acid can react with the N-terminal amine of KR-12 to form a standard amide bond. Grafting gallic acid (3,4,5-trihydroxybenzoic acid) to KR-12 produced GA-KR12, a dual-action peptide. Chu et al. reported a significant decrease of *Streptococcus mutans* mono-species in biofilms [102]. It also prevented the demineralization of tooth hard tissue and enhanced the remineralization of artificial caries on enamel and dentine.

Fatty acids with varying chain lengths are frequently utilized to improve peptide properties. These peptide conjugates have the family name lipopeptides. Lipopeptides form a special class of natural peptides because the acyl chain can confer aggregation property to the peptide by forming micelles, which fall into the category of nanomaterials. They are apparently important since multiple lipopeptide antibiotics, including daptomycin, colistin, telavancin, oritavancin, and dalbavancin, are in clinical use. Kamysz et al. [49] conjugated different n-alkyl acids (C2, C4, C6, C8, C10, C12, and C14) with the N-terminus of KR-12. This conjugation decreased one positive charge and increased hydrophobicity of the peptide. KR-12 conjugated with the C8 fatty acid showed the highest antimicrobial activity against a panel of Gram-positive and Gram-negative bacteria, including *S. aureus*, *Enterococcus faecium*, *K. pneumoniae*, *A. baumannii*, *P. aeruginosa*, and *Klebsiella aerogenes* (MIC 1–4 μg/mL). However, KR-12 became less active when conjugated with either shorter or longer fatty acids. Moreover, peptides coupled with C10-C14 fatty acids are highly hemolytic with 10% hemolysis concentration (HC_10_) below 6 μg/mL. This work indicates the importance of fatty acid chain length for optimal peptide performance.

Lei et al. designed Myr-KR-12N and Myr-KR-12C by coupling a myristoyl group (C14) with the N and C-terminus of KR-12, respectively. Both peptides exhibited broad-spectrum and intensified bactericidal activity by disrupting bacterial cell membranes. These derivatives displayed remarkable ability to spontaneously assemble into nanoparticles when mixed with deionized water [103]. The myristoylated KR-12 nanobiotic possesses significant LPS binding capacity and effectively reduces inflammation in vitro. In addition, Myr-KR-12N showed superior capability to rescue mice from lethal *E. coli*-induced sepsis in comparison with the conventional antibiotic meropenem.

### 4.2. Sidechain-Linked Peptides: Stapled KR-12

In 2024, Zhang et al. applied staples to KR-12 to stabilize the peptide helical structure [55]. Gregory Verdine first utilized Ruthenium-mediated olefin metathesis chemistry, developed by Grubb, to establish hydrocarbon bridges between adjacent sidechains to stabilize the helical structure [104]. Creation of staples in peptides provides an effective method to stabilize peptides since the construction of two such staples in the HIV fusion inhibitor enfuvirtide enabled oral availability of this man-made antiviral drug [105]. A particular staple may only stabilize a local structure. Moreover, the optimal staple positions are largely unknown and usually a systematic scan is conducted to identify the best candidate [106,107]. In the case of KR-12, it was found that the (i, i+7) type staples, with two forms of products, did not bring forth potent peptides [55], whereas the (i, i+4) type bonding was effective. One such staple made KR-12 more helical in 50% TFE and less susceptible to protease digestion. The peptide became more helical and stable with the shift of the staple from the N-terminus to the C-terminus, resulting in a rise of half-life (t_1/2_) in human serum from 2.38 h to 18.28 h. Within a series of (i, i+4) staples, the (Q22, D26) bridge is most effective in conferring activity to KR-12 (Table 1). In the NMR structure of KR-12 (Figure 3C), the hydrophilic sidechains of both Q22 and D26 approach each other to favor hydrogen bond formation [32]. Therefore, the Q22-D26 staple would stabilize the helix in a similar manner. Another advantage of the (Q22, D26) staple is that it removed the negative charge of D26 and increased the net charge of the peptide by +1, increasing antibacterial activity. In contrast, peptides with two basic amino acids stapled lose two positive charges and became more hydrophobic, leading to undesired toxicity to host cells. The increase in peptide hydrophobicity is most evident each time when such a staple involves either interfacial R23 or K25. In addition, the R19–R23 staple strained the critical R23 from interaction with bacterial membranes, leading to an inactive peptide [55]. Hence, our KR-12 structural knowledge [32] shone light on these constructs. Such staples have also been applied to other AMPs, including Lasioglossin-III, melectin magainin, aurein 1.2, and anoplin [108,109,110,111].

### 4.3. Head-to-Tail Cyclization of KR-12

Another approach for improving peptide pharmacokinetics (PK) is to design cyclic KR-12. Cyclic antimicrobial peptides formed via a peptide bond between the N-terminus and C-terminus are also called circular peptides. Such peptides have been discovered in bacteria, plants, and animals [112,113]. The design of cyclic KR-12 was inspired by plant cyclotides [112]. In the initial design, Gunasekera et al. connected two copies of KR-12 with short linker sequences (CGG or GAGG) [114]. After cleavage and purification, a linear peptide with glycine and cysteine at termini can then be cyclized by using the native chemical ligation [115]. The effects of the linker length (2, 3, and 4 residues) on MICs of dimeric peptides cd2, cd3, and cd4 were minimal. It is also possible to reverse the sequence of one copy (e.g., retro-cd2) or both copies (2retro-cd4) of KR-12 in the design [115]. Interestingly, the linear dimeric forms are equally active, indicating cyclization is unimportant for peptide activity. All the designed dimeric constructs demonstrated activity comparable to LL-37 but eight- to sixteen-fold stronger than KR-12. Enhanced antimicrobial activity is proportional to increased membrane permeabilization of liposomes. Due to signal overlapping in NMR spectra, the authors used CD to confirm the helical structure of the cyclic construct when bound to SDS or lysophosphatidylglycerol (lysoPG) micelles. Since this dimeric form possesses a wider hydrophobic surface, it showed increased cytotoxicity as well. Most importantly, cyclic peptides with a longer linker (e.g., cd4) demonstrated enhanced peptide stability in 25% human serum. While the linear form of peptides such as LL-37 and KR-12 was degraded rapidly, 50% cd4 and cd4(Q5K,D9K) were detected after 6 h.

Subsequently, the two copies of KR-12 in the cyclic peptide were also cross-linked via a disulfide bond to further improve peptide stability [116]. The dimeric peptide with a GAGG linker is referred to as cd4-CC, while the cd4-CCPP construct contains a proline in the linker (GPGG). The incorporation of a proline doubled bactericidal activity, probably due to increased helicity. While human LL-37 retains anti-*E. coli* activity in the presence of different types of salts (except for CaCl_2_), antibacterial activity of KR-12 is sensitive to NaCl [117]. LL-37 loses its antibacterial activity in the presence of 25% human serum due to binding to human apolipoprotein A-I [118]. Remarkably, cyclic peptides cd4-CC and cd4-CCPP (MIC 0.312-0.625 µM) retains much activity against both *E. coli* and *P. aeruginosa* in the presence of salts or human serum (MIC 0.625-5 µM). These results underscore the therapeutic potential of the cyclic form of KR-12. A new cyclic KR-12 peptide dubbed CD4-PP (Table 1) was also synthesized recently [52]. LL-37 is known to be cleaved by the *S. aureus* protease aureolysin [42]. CD4-PP gained stability to this protease with 20% detected after 6 h incubation, while the control LL-37 was nearly completely degraded in 1 h. CD4-PP with two symmetric copies of KR-12 (Q22K and D26A) (Figure 4L) is potent and can reduce the *E. coli* attachment to urinary catheters and substantially reduce uropathogenic *E. coli* infection. These exciting results opened great opportunities for the medical applications of these novel cyclic KR-12 constructs. 

### 4.4. Surface Immobilized KR-12

Many people use medical implants at least once in their lives. One of the major issues is the potential for bacterial infection after surgery. Such bacterial infections are usually in the form of biofilms, and therefore extremely challenging to remove [119,120]. In addition, conventional antibiotics became ineffective due to antibiotic resistance. While the ultimate goal is to get rid of implant replacement, two strategies can be taken to handle such implant infections. The first is to treat the infection with effective antimicrobial peptides. We demonstrated this treatment option with 17BIPHE2, a potent, selective, and stable peptide designed based on GF-17 (Figure 2C), the major antimicrobial peptide of LL-37 [121]. Another LL-37-engineered, slightly longer peptide SAAP-148 (Figure 2C) can eliminate persisters of MRSA as well [122]. The second strategy is to coat the medical implants with AMPs. Both human LL-37 and FK-16 have been covalently immobilized onto a titanium surface [123,124]. Covalent coupling achieved via the click chemistry of thiol–maleimide reaction provides the advantage of long-term stability to avoid peptide leaching. The FK-16 coated surface can effectively kill the ESKAPE pathogens [124]. KR-12 has also been chemically coupled to the titanium surface via a slightly different chemistry where the carboxylic group of D26 of KR-12 formed an ester bond with amine of the 3-(2-aminoethylamino) propyltrimethoxysilane linker [45]. This sidechain-coupled KR-12 showed activity against *E. coli* (MIC 16 µg/mL), *S. aureus* (MIC 64 µg/mL), and *S. epidermidis* (MIC 32 µg/mL). The KR-12 coated titanium surface effectively decreased bacterial adhesion and promoted osterogenic differentiation of human bone marrow mesenchymal stem cells (hBMSCs). Using yet another different procedure, Kozuka et al. immobilized KR-12 by dipping and coupling using a polyethylene glycol (PEG) linker [125]. The resulting surface was found to be active against *E. coli* with reduced live bacteria but less effective against Gram-positive *S. aureus*. Such an activity spectrum of the coated KR-12 is similar to that of the original KR-12 in the free form [32].

Chanci et al. investigated surface absorption and immobilization on peptide activity [64]. While free KR-12 inhibited *S. aureus* ATCC 25923, it lost activity in the physisorbed or covalently bonded states. This is in contrast to the surface immobilization results above where KR-12 retained activity. The most probable reason is the direct immobilization of KR-12 via amine of the peptide N-terminus, K18 or K25, without using a flexible linker. Hence, this fixed KR-12 can neither move toward bacteria nor change its structure to a helical conformation to damage membranes. Combined, these KR-12 studies reinforce the importance of flexible linker for orientational immobilization of KR-12 to endow activity to biomaterials.

### 4.5. Peptide Formulation

Formulating new or existing drugs into different dosage forms can modify pharmacokinetic events to meet pharmacodynamic endpoints, thereby achieving therapeutic benefits [126]. Sustained release formulations can prolong the release of the active pharmaceutical ingredient over an extended period, reducing either the dose or dosing frequency. Delayed-release formulations can delay drug release at harsh absorption sites until it reaches a more favorable one. The nano-formulation of peptides has been identified as a promising approach for improving the stability of peptides and providing metabolic stability and bioavailability [127,128]. Formulations may also reduce peptide potential toxicity and make peptide stability less demanding [129,130].

Wound dressings play a crucial role in accelerating the wound-healing process. Various biocompatible materials, including silk, gelatin, cellulose, chitosan, alginate, polyurethane, poly(lactide-co-glycolide), polyvinyl alcohol, and poly(ε-caprolactone), are commonly used. However, these materials often fail to prevent microbial colonization. Incorporating antimicrobial agents directly into these materials offers a solution to eliminate pathogens in chronic wounds and enhance healing. Su et al. demonstrated the elimination of bacterial burden in wounds using the nanofiber/17BIPHE2 dressing [129]. Alternatively, AMPs can be immobilized to dressing materials. Using thiol–maleimide click chemistry, Song et al. [59] immobilized Cys-KR-12 (seq: CKRIV**K**RIK**KW**LR) to electrospun silk fibroin nanofiber membranes covalently. Three sites in this KR-12 form were altered to enhance peptide activity: Q22K, D26K, and F27W. In addition, a cysteine at the N-terminus is added to facilitate the click chemistry between the peptide thiol and maleimide. This functionalized nanofiber coated with high-density KR-12 can eliminate Gram-positive bacteria *S. aureus*, *S. epidermidis*, Gram-negative bacteria *E. coli*, and *P. aeruginosa*. This nanofiber can be stored for at least three weeks at 4 °C without losing activity. This immobilized KR-12 also inhibits biofilm formation, promotes cell differentiation, and suppresses inflammation, features desired for wound healing.

Blasi-Romero et al. covalently immobilized KR-12 to wood-derived cellulose nanofibrils (CNFs) which can provide a favorable environment for chronic wound healing in terms of moisture and reepithelialization [131]. Different chemistries, including amine coupling through carbodiimide chemistry, thiol–ene click chemistry, and Cu(I)-catalyzed azide–alkyne cycloaddition, were compared and the thiol–ene click chemistry was found to be most effective in endowing antibacterial activity against *E. coli* and anti-inflammatory properties to CNFs. The poor activity of the immobilized KR-12 against *S. aureus* is reminiscent of the observation with free KR-12 (see above), which failed to inhibit the growth of this bacterium like its parent peptide LL-37 in rich media [32].

Conjugation of KR-12 with cell-penetrating peptides (CPP) can increase the killing of intracellular pathogens. CPP-KR12@Si was developed using biomimetic silica precipitability to form silica particles with self-entrapped CPP-KR12 peptide [53]. Microdilution antibacterial assays found that CPP-KR12 is much more active than KR-12 itself. Formulation alters peptide activity depending on both bacteria and peptide. This formulation enables controlled peptide delivery to bacteria and cells, improves stability against trypsin, and reduces cytotoxicity to Raw264.7 cells. However, hemolysis increased after formulation due to the toxicity of silica (SiO_2_) particle itself. In addition, CPP-KR12 combined with a bone graft substitute (BGS) was demonstrated to be safe. Therefore, this approach is promising for developing safe and effective drug–device combination products for tissue regeneration [53].

## 5. Beyond KR-12: Discovery and Design of Even Shorter LL-37 Peptides

### 5.1. Short Lipopeptides Designed Based on KR-12 Segments

**Figure 6:**
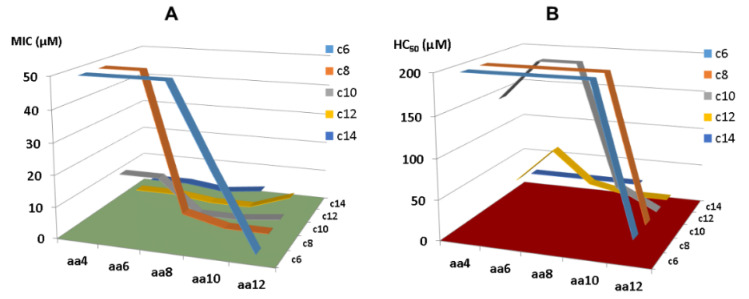
2D array of the lengths of the KR-12 peptide with 4 to 12 amino acids (aa4 to aa12) and fatty acids (c6 to C14) for antibacterial activity (**A**) and hemolytic toxicity (**B**) uncovered a zone for designing selective lipopeptides. On the left, the closer the curves to the green plane, the more potent the peptides are. In contrast, the farther away from the red plane on the right, the less hemolytic the peptides are.

Conjugation provides a practical avenue to even shorter KR-12 peptides. Owing to the desired properties of artificial short lipopeptides we observed [132], we subsequently made a library of peptides and searched for even shorter LL-37 peptides via systematically conjugating C4-C14 fatty acids with KR-12 segments with 4, 6, 8, 10, and 12 amino acids [51]. In addition, V21 was altered to W for peptide quantification. While short KR-12 segments (KR4, KR6, and KR8) plus C6 fatty acids are not antibacterial, long KR-12 segments (KR-10, KR-12) in conjugation with long fatty acids (C12 and C14) lead to active peptides with high toxicity. There is a preferred zone where the designed peptides are both potent and selective (Figure 6). Within this zone, we identified two outstanding lipopeptides, C10-KR8 and C8-KR10, which are potent and selective. When D-amino acids were incorporated, the resulting lipopeptides (C10-KR8d and C8-KR10d) gained stability to proteases as well. Importantly, proteomic studies further revealed the merits of C10-KR8d. This D-peptide has a poor ability to associate with numerous serum proteins compared to the L-form made of natural amino acids. This is understandable since the D-form with an opposite chiral configuration does not fit well into the active site of proteases. In addition, C10-KR8d retains activity under different media conditions, such as salt, pH, and serum, leading to an attractive candidate for in vivo studies [51].

### 5.2. Design of LL-37mini Based on KR-8 Identified in Diluted Media

As some AMPs can better preserve activity in diluted MHB, we screened a series of small overlapping fragments covering the entire sequence of LL-37 (≤10 amino acids) [83]. Both the N-terminal and C-terminal small fragments of LL-37 (LL-10 and LR-10) do not inhibit bacteria. It is important to note that these peptides are inherently non-antibacterial and activity is not created in the diluted media. However, KR-8 and RIK-10 are active in 12.5% MHB. Importantly, RIK-10 remains active even in normal MHB or Dulbecco’s modified Eagle medium (DMEM), identifying yet another small active peptide within LL-37 [83]. Interestingly, both KR-8 and RIK-10 peptides are derived from FK-16, the major antimicrobial region of LL-37 (residues 17–32). Different from these short peptides, the antibacterial activity of FK-16 remains essentially the same in 12.5% MHB or standard MHB (100%). Likewise, multiple antibiotics did not change their activity with and without media dilution [83]. Clearly, the activity of antimicrobial agents is not compromised when they do interact with media components.

Using KR-8 as the shortest template of LL-37 (residues 18–25), we designed a series of peptides. Tryptophan (W) was incorporated since it has a bulky hydrophobic side chain preferring the membrane interface [133,134,135]. In addition, we inserted arginine into the peptide due to its preferred association between W and arginine in Trp-rich peptides [136]. In a family of designed ultrashort peptides, the LL-37mini (RRWWRWWR-amide) has multiple desired properties. First, it rapidly killed *S. aureus* USA300 and its anti-MRSA activity is minimally affected by both acidic pH and physiological salts. Second, it is non-toxic to human red blood cells (RBC) and HaCaT skin cells. Third, MRSA did not develop resistance to LL-37mini after multiple passages over 17 days, probably due to its membrane targeting. Finally, it exhibited excellent antibiofilm activity in a murine wound model comparable to daptomycin [83]. Further comparative studies are required in order to know how closely LL-37mini peptides mimics LL-37 functions.

## 6. Safety and Efficacy of KR-12 Engineered Peptides in Animal Models

**Figure 7:**
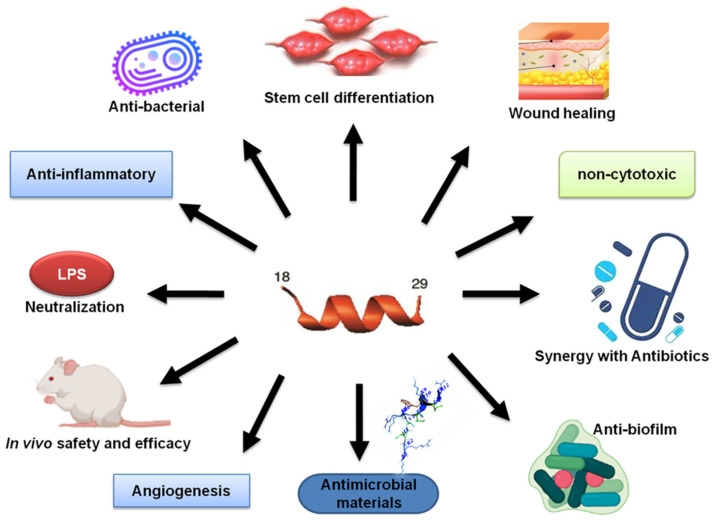
Like LL-37, KR-12 peptides also possess numerous desired properties such as antimicrobial, antibiofilm, and LPS neutralization. KR-12, as well as LL-37, has been covalently immobilized onto titanium (Ti) implants [123,124].

Animal models are significant in assessing biosafety and peptide efficacy of antimicrobial peptides, allowing a detailed understanding of whether these strategies are appropriate for the translation from bench to bedside. However, no ideal animal model exists for assessing the efficacy and toxicity of antimicrobial peptides for various bacterial, fungal, and parasitic infections. According to the APD [137], at least 180 AMPs have been evaluated in animal models, including both invertebrate (e.g., insects) and vertebrate organisms (e.g., mice). Most of the studies utilized murine models (68%). KR-12 has been demonstrated to have antibacterial, antifungal, antiparasitic activity, anti-LPS, and immunomodulatory properties [32,116,138]. Its engineered constructs (Figure 5) are anticipated to be more potent for treatment. A translational infection methodology and a size adequate to the requirements of the experimental design are greatly envisaged. In diverse in vivo applications, studies have revealed promising results for the KR-12 peptides (summarized in Table 2). As discussed in the previous section, this 12-residue peptide mimics LL-37 in numerous ways (Figure 7). Some of these functions have been observed in animal models (below).

### 6.1. In Vivo Safety Evaluation

#### 6.1.1. Systemic Toxicity

To assess the safety of the nanobiotics Myr-KR-12N and Myr-KR-12C in vivo, the authors conducted experiments using mice as follows: Initially, five mice were intraperitoneally (i.p.) injected with Myr-KR-12N nanobiotic (100 µg per mouse) or a vehicle, and their body weight was monitored over a 7-day period. No significant differences in body weight were observed during this duration. To further evaluate the in vivo toxicity of these peptides, four groups of mice (with six mice per group) were subcutaneously injected with either 240 µg of KR-12, Myr-KR-12N, or Myr-KR-12C, or PBS every two hours, with a total of six administrations, following a previously established protocol [147]. Twenty hours after the final injection, liver, kidney, and serum samples were analyzed for liver function (aspartate aminotransferase (ALT) and alanine aminotransferase (AST)) and kidney function (blood urea nitrogen (BUN) and creatinine (CREA)). Additionally, the study revealed no biochemical or histopathological abnormalities in the KR-12, Myr-KR-12N, or Myr-KR-12C groups. These in vivo studies demonstrated low toxicity of Myr-KR-12N and Myr-KR-12C, thereby suggesting their potential for clinical translation [103].

#### 6.1.2. Topical Ototoxicity of KR-12-a2 to Guinea Pigs

Sung et al. studied MRSA, a rising concern for treating chronic suppurative otitis media and pediatric tympanostomy tube otorrhea [139]. In this study, authors evaluated the ototoxicity of KR-12-a2 (sequence 42 in Table S1) by applying the solution topically for seven days in the middle ears of guinea pigs. Ototoxicity was assessed by auditory brainstem evoked response and scanning electron microscope examination. It was found that at 50 µg of KR-12-a2, the hearing threshold was similar to PBS control. SEM findings show that the KR-12-a2 group has intact outer hair cells, proving that the peptides do not display any adverse hearing damage upon topical application. Further, in vivo studies are crucial to evaluate topical efficacy of KR-12-a2 by establishing MRSA otitis animal model [139].

### 6.2. In Vivo Efficacy Evaluation of KR-12 Peptides in Topical and Systemic Animal Models

#### 6.2.1. Catheter Associated Bacterial Biofilm Mice Model

Biofilms can often form in catheters and are difficult to remove. Using a murine biofilm model [148], we evaluated the efficacy of C10-KR8d in preventing biofilm formation. The peptide could eliminate MRSA effectively in catheters and surrounding regions [51], while a peptide inactive against MRSA was unable to reduce bacterial burden [121]. Meanwhile, we detected upregulation of MCP-1/CCL2 and IL-17A on day 3, which recruit monocytes and neutrophils to further clear the MRSA infection. After 17BIPHE2 treatment, we previously noticed also on day 3 an increase in CXCL-10 (an antimicrobial peptide) that exerts a second hit on bacteria [121].

#### 6.2.2. Wound Healing Models

The murine wound healing model has been widely utilized to demonstrate antimicrobial activity of AMPs. LL-37 is known to play a role in wound healing [149]. Liu et al. [140] constructed a multifunctional cryogel (HA/TA2/KR12) consisting of hyaluronic acid (HA), tannic acid (TA), and KR-12. A modified version of KR-12 (Seq: CKRIVKRIKKWLR) was used to confer antimicrobial potency as well as anti-oxidation ability (via N-terminal cysteine). Zhang et al. [141] incorporated KR-12 into regenerated silk fibroin (RSF) films to achieve antibacterial and anti-inflammatory effects. Recently, we showed that KR-8-derived LL-37mini was also able to reduce MRSA burden in chronic mouse wounds [83].

Q22-D26-stapled KR-12 was shown to be more effective than LL-37 in reducing *E. coli* burden at day 7 in murine wounds [55]. In addition, this engineered KR-12 treatment increased IL-6 and TNF-α on day 7 and decreased their levels on day 14. This implies an initial proinflammatory effect followed by anti-inflammation on day 14, presumably to promote wound healing. Surprisingly, LL-37 did not show any effect in this animal model. However, LL-37 worked in a manner similar to the stapled KR-12 in a murine macrophage stimulated with 200 ng/mL LPS.

#### 6.2.3. LPS-Induced Bone Erosion in Mice Models

Li et al. established a mouse model of LPS-induced inflammatory bone loss by stimulating distinct bone erosion in the spongiosa of murine femurs [142]. Five-week-old C57BL/6 mice received two doses of LPS intraperitoneally (5 mg/kg). Post LPS injection, the animals were treated with KR-12-a5 (2 mg/kg) every alternate day for seven days. The femur high-resolution micro-computed tomography (micro-CT) analysis revealed increased bone/tissue volume, trabecular number, trabecular separation, and connectivity density. Further KR-12-a2 restored the inflammation-caused bone loss and enhanced collagen expression. This effect resulted from increased redifferentiation of bone marrow stem cells [142].

#### 6.2.4. Evaluation of Osteointegration of Surface Immobilized KR-12 in Rat Models

PEEK (polyetheretherketone) is a potential bone implant material for its outstanding biocompatibility and elastic modulus, similar to natural bone [144]. However, due to bioinertness, the PEEK lacks osseointegration and antimicrobial properties. With the aid of polydopamine (PDA), Meng et al. studied the effects of KR-12 coating on bone formation by implanting different materials into rats: PEEK, PEEK-PDA, and PEEKPDA-KR-12. Micro-CT images revealed that implants with KR-12-PDA coating had the highest bone volume around them three months after implantation in rat femurs. Analysis of bone volume fraction (BV/TV), bone mineral density, trabecular number, and trabecular thickness indicated superior bone regeneration in the PEEKPDA-KR-12 group. Polarized light and staining techniques showed that PEEK-PDA-KR-12 implants were better integrated with bone tissue than unmodified ones. Higher hydrophilicity and Ca^2+^ ion deposition were observed in the PEEK-PDA-KR-12 group, contributing to increased tissue compatibility and osteogenesis. Results from in vitro studies were confirmed in vivo, with PEEK-PDA-KR-12 implants demonstrating excellent cytocompatibility, osteogenic differentiation, and outstanding osteogenesis. The study suggests that surface modification with PDA-KR-12 enhances the osteogenesis properties of implants, leading to improved in vivo performance compared to unmodified counterparts. These findings highlight the significance of surface modification for enhancing bone-implant integration and tissue compatibility [144].

#### 6.2.5. Induced Colitis in a Mouse Model

Inflammatory bowel diseases (IBD) encompass a collection of persistent gastrointestinal tract disorders characterized by a multifaceted origin, with intestinal dysbiosis standing out as a primary contributor. Fabisiak et al. evaluated the anti-inflammatory and antibacterial properties of the human cathelicidin LL-37 and KR-12 in colitis mouse models [145]. The animal used two colitis inducing chemicals: (1) 2,4,6-trinitrobenzenesulfonic acid (TNBS) for acute, chronic, and semi-chronic condition and (2) dextran sulfate sodium (DSS) for semi-chronic condition in mice. In the chronic colitis model, the mice group treated with KR-12 intraperitoneally and twice daily (BID) (1 mg/kg, BID) showed an anti-inflammatory benefit and demonstrated a statistically significant reduction of tissue damage compared to LL-37 treated mice. Moreover, in the semi-chronic mice group treated with KR-12 at 5 mg/kg, i.p., BID, the authors observed decreased intestinal damage compared to TNBS-treated mice. Significantly, in the semi-chronic DSS-induced colitis model, KR-12 treatment led to a decrease in *E. coli* and coli group, opening a novel avenue to restoring the microbiota for IBD patients [145]. Since KR-12 is a narrow-spectrum antimicrobial peptide and not toxic [32], this study beautifully illustrates the great potential of KR-12 as a novel anti-inflammatory and microbiota-restoring therapeutic.

#### 6.2.6. *E. coli*-Induced Lethal Sepsis in a Murine Model

Motivated by the commendable anti-inflammatory effects observed in vitro studies, Lei et al. [103] proceeded to induce endotoxin sepsis in a murine model through intraperitoneal co-administration of LPS (10 ng/mouse) and D-galactosamine (18 mg/mouse), a substance that heightens animals’ sensitivity to LPS. In the control group treated with a placebo, 90% of the subjects succumbed within 24 h post-LPS injection. The application of 10 µg KR-12 or meropenem per mouse did not significantly enhance the survival rate, whereas the survival rate reached 100% following treatment with an equivalent dosage of Myr-KR-12N, Myr-KR-12C, or polymyxin-B (PMB). Lei et al. [103] further evaluated the LPS neutralization capabilities of these peptides by elevating the LPS dose to 50 ng per mouse. The administration of 10 µg of Myr-KR-12N, Myr-KR-12C, and PMB per mouse rescued all septic mice. To delve deeper into the efficacy of anti-endotoxic sepsis among Myr-KR-12N, Myr-KR-12C, and PMB, an even higher LPS dose of 1 µg per mouse was introduced. While PMB rescued one mouse out of ten experimental animals, Myr-KR-12N saved eight out of ten septic mice. In contrast, no rescuing efficacy was observed when Myr-KR-12C was administered at 1 µg per mouse. These findings uncover that myristoylated KR-12 nanobiotics, especially Myr-KR-12N, can significantly mitigate endotoxin-induced immune responses. Moreover, Myr-KR-12N surpasses PMB in terms of LPS neutralization efficacy.

#### 6.2.7. Systemic Efficacy of Lipopeptide C10-KR8

Because our engineered peptide C10-KR8 crossed a series of in vitro hurdles (Section 5.1), we tested its systemic efficacy in neutropenic mice, which are widely utilized for this purpose. A standard protocol was used to produce neutropenic mice via two cyclophosphamide injections prior to infection [150,151]. In our C10-KR8d study [51], we infected the animals via intraperitoneal injection and started to treat the infected mice two hours post infection. Our treatment dose was found based on the maximum tolerated dose (MTD) of 20 mg/kg. A statistically significant bacterial burden drop was observed in lung and liver when treated at 5 mg/kg, establishing a positive efficacy for this KR-12-derived lipopeptide. This observation motivated us to develop LL-37 mimicking peptides with improved systemic efficacy. Both horine and verine demonstrated exceptional efficacy when administered via either i.p. or intravenous (i.v.) route [152].

**Figure 8:**
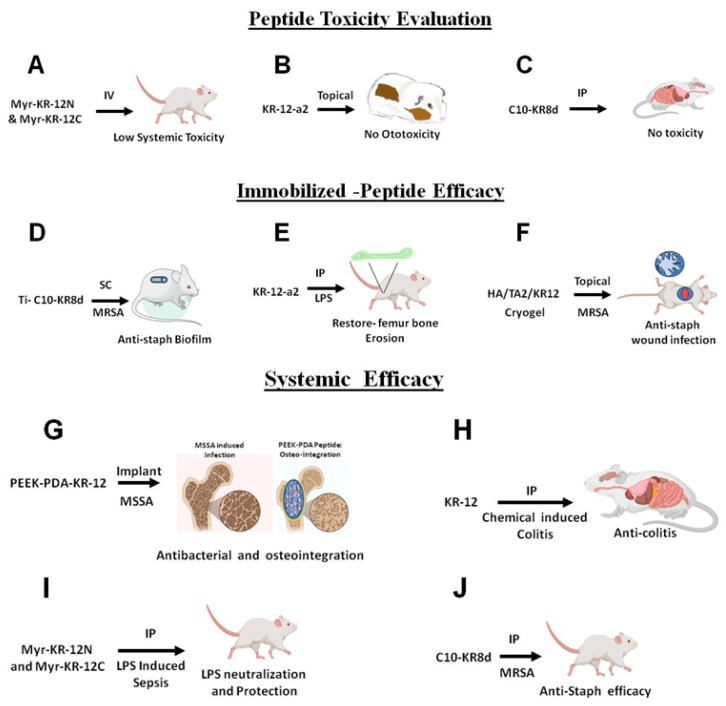
In vivo safety and efficacy of KR-12 constructs. (**A**) Systemic toxicity of nanobiotics Myr-KR-12N and Myr-KR-12C via intravenous administration in mice. (**B**) Ototoxicity of a KR-12-a2 by applying the solution topically into the middle ears of guinea pigs. (**C**) Identification of the non-toxic dose of C10-KR8d via the intraperitoneal route in mice. (**D**) Efficacy of Ti-C10-KR8d implant on catheter-associated MRSA biofilm in mice. (**E**) LPS neutralization and bone restoration efficacy of KR-12-a2 in mice. (**F**) Cryogel-HA/TA/KR12 topical application in a mouse wound model. (**G**) PEEK-PDA-KR-12 coating on implants shows both antibacterial and osteointegration potential in mice. (**H**) KR-12 has anti-colitis ability against chemical induced colitis in mice. (**I**) Myr-KR-12N and Myr-KR-12C protection from LPS sepsis in mice. (**J**) C10-KR8d showcased anti-MRSA efficacy in a neutropenic murine infection model.

In summary, both toxicity and efficacy have been evaluated for select KR-12-derived constructs using a variety of animal models (Figure 8). These studies laid a foundation for future developments.

## 7. Conclusions and Perspectives

As a Chinese traditional art, a piece of paper can be folded into a variety of shapes. Herein based on the KR-12 peptide template first reported by Wang in 2008 [32], a variety of constructs (origami) have been created (Figure 5). Due to its small size, selectivity, narrow-spectrum activity, and LPS binding capability, this small LL-37 fragment has attracted worldwide attention, leading to a panel of innovative engineering strategies for KR-12, spanning from simple amino acid substitutions, peptide conjugates to complicated stapled peptides and head-to-tail cyclized forms. From these studies, we have achieved the following:

1. As the smallest antibacterial peptide, the antibacterial activity of KR-12 is very susceptible to chemical modification. Even terminal capping has an evident effect on peptide activity. Our media dilution has deepened our understanding of the activity differences of these peptides reported from different labs [83]. To facilitate activity comparison from different laboratories, it is best to conduct antimicrobial assays by following the Clinical and Laboratory Standards Institute (CLSI) standard procedure. However, one may use a more sensitive media condition in initial peptide screening to identify more antimicrobial templates. Under such a condition, we identified KR-8 and RIK-10, two active peptides smaller than KR-12. These minimized LL-37 peptides enriched our antimicrobial reservoir.
2. Peptide conjugation can enrich the structure and function of KR-12, including cell penetration, carbohydrate binding, and structural stabilization [48,50,54]. KR-12 can also be conjugated with fatty acids, providing another route to selective and potent peptides shorter than KR-12. As a special conjugation, KR-12 has been covalently immobilized via different chemistries onto biomaterials to confer antimicrobial and anti-inflammatory activities. In the presence of a flexible linker, the activity spectrum of free KR-12 is maintained.
3. The first common theme in KR-12 engineering is to enhance peptide activity [39,85]. For this purpose, both basic and hydrophobic amino acids are increased. The 3D structure of KR-12 in complex with D8PG [32] and SDS [115] laid a solid basis for peptide design. Q22 and D26 are most obvious and logical to change. Evidently, peptide toxicity sets a limit to these changes. The increase in peptide toxicity is more pronounced, however, when interfacial R23 and K25 of KR-12 are converted to a hydrophobic leucine [80]. It is an art to control peptide hydrophobicity to the right level so that the peptide construct retains potency and lacks toxicity [12,35]. Similarly, one cannot keep increasing basic lysine/arginine since toxicity also rises once a certain limit is passed [153].
4. Another desired goal is to confer stability to KR-12 to increase its bioavailability. Both simple and complex methods have been utilized. Simple practices include C-terminal amidation, N-terminal acetylation, D-amino acid incorporation (partially or in full), and lipidation, whereas conjugation with a Trp-cage, creation of hydrophobic staples and head-to-tail cyclization are more complex engineering practices. Most of the more stable forms of KR-12 appeared after 2020 (see timeline in Table 1). While lipopeptides such as C10-KR8d gains stability by incorporating D-amino acids [51], sidechain-linked staples stabilize the helical structure of KR-12 to diminish protease digestion [55]. Inspired by natural cyclotides, the engineered cyclic KR-12 construct becomes more stable because of unexposed termini and structural stabilization [114,116]. One possible reason for the dimeric design is that direct head-to-tail cyclization of a short LL-37 fragment may not be feasible due to the steric strain in the cyclic structure. Formulation of KR-12 in polymers can also protect the peptide from degradation [130].
5. Because LL-37 is known to be inactivated by human serum [118], a third important goal of KR-12 engineering is to increase peptide bioavailability. Conversion of L-amino acids of C10-KR8 to D-amino acids provides a classic but useful strategy. With a different chiral configuration, the association of C10-KR8d with proteases and other proteins is minimized [51]. The cyclic peptide also retained activity in serum [116]. In addition, multiple KR-12 constructs have shown in vivo efficacy in topical models (catheter or wound healing) (Table 2). These innovative KR-12 constructs have enriched our LL-37 based defense arsenal.
6. Mechanistically, KR-12 with a minimal antibacterial sequence can cluster anionic lipids and permeabilize bacterial membranes similar to its parent peptide LL-37 [43,75,154]. Most of the enhanced KR-12 peptides discussed herein can also damage bacterial membranes [43,55,116]. Recently, attention has also been paid to peptide amyloid formation, which is proposed to play a role in bacterial killing. Although oligomers (α-amyloids?) have been detected for KR-12, FK-13/LL-37(17-29) [85,86], and LL-37, all helical peptides [65,89,90,91], further studies are required to determine to what extent such a process contributes to bacterial killing.
7. We should not forget that bacteria can respond to LL-37 and its peptides in numerous ways, including membrane modification to diminish peptide killing [10]. For this purpose, bacterial transposon libraries and proteomic studies [155,156] provide powerful tools for mapping bacterial resistant genes. However, we should emphasize that LL-37 peptides, including the KR-12 constructs engineered and discussed herein, remain potent against drug-resistant pathogens, persisters, and their biofilms [51,52,121,122]. Bacteria did not develop resistance to KR-12-derived peptides such as C10-KR8d or LL-37mini in multiple passage experiments [51,83].

Although a challenging task [157], further preclinical studies of these exciting KR-12 constructs may move these KR-12 constructs to clinical trials. These include minimization of peptide cost via large-scale production, minimization of peptide toxicity, maximization of in vivo efficacy, application scope of each construct, and regulation issues for drug approval.

1. Peptide cost and large-scale production: It is critical to address peptide synthesis, purification, and large-scale production challenges [158]. While small peptides such as C10-KR8 with only eight residues (Table 1) can take full advantage of the existing large-scale industrial peptide synthesis capability, longer constructs such as KR-12 conjugated with Trp-cage (Table 1) may be produced via fermentation. However, we may not be able to produce cyclic dimeric KR-12 entirely by fermentation until the last step of the head-to-tail cyclization can be accomplished enzymatically. The scalability is equally important for formulated or surface immobilization KR-12. In addition, one should avoid activity loss due to chemical modification during storage of a large quantity of highly purified peptide therapeutics. Some AMPs can lose activity due to oxidation (e.g., methionine) [159,160], while succinimide (intramolecular cyclization of the sidechain of aspartic acid with its backbone amide) KR-12 showed reduced activity, helicity, and serum stability [161].
2. Safety: Toxicity studies of KR-12 constructs are very limited and more thorough evaluations are needed both in vitro (using different cell lines) and in vivo (two animal models, per U.S. Food and Drug Administration (FDA) regulation). This safety requirement need be extended to chemicals used for formulation. The use of FDA-approved formulants is a shortcut. The safety evaluation may include the potential impact of new drugs on microbiomes as well.
3. Efficacy in animal models: Continued studies are required to evaluate the efficacy of various constructs of KR-12 in proper animal models that mimic different diseases. Rigorous preclinical studies are necessary to evaluate PK/pharmacodynamics (PD) and biodistribution of KR-12 constructs in animal models and, eventually, in humans. Age and sex differences to treatment outcomes should be considered. Similar studies on formulated KR-12 are also needed. Ultimately, one may also make use of other cell models or even newly developed AI algorithms for efficacy and safety evaluation without the use of animals [162].
4. Application scope: Future studies may find practical applications for various engineered constructs of KR-12 described herein and to be engineered. Depending on the problem, a proper construct may be chosen. For the sake of cost, one would embrace the simplest molecular design. When KR-12 constructs alone do not produce the desired treatment outcomes, combination treatment may be considered [140,163,164]. In addition, formulations can expand their application scope. Natural vehicles such as exosome-loaded LL-37 have recently been reported to protect against Zika virus infection [165]. It is exciting that precise nanopores produced via 3D printing can also work synergistically with LL-37 mimicking peptides to promote wound healing [166]. These delivery vehicles may increase the therapeutic efficacy of AMPs and their inducers if sufficient attention can also be paid to safety.
5. Regulatory and clinical pathways to novel peptide antibiotics: Understanding the regulatory requirements and clinical development pathways for KR-12 constructs in particular and antimicrobial peptides in general, including formulations and immobilization, will facilitate their translation from laboratory to clinical settings. Collaborative efforts among researchers, industry, and regulatory agencies are required to streamline the development, to establish proper standards for peptide drugs, and to provide minimal equipment for transporting and storing peptide drugs.

## Figures and Tables

**Table 1:** Timeline of select KR-12-derived antimicrobial peptide constructs ^1^.

Year	Name	Amino Acid Sequence	Comments	Refs.
2008	KR-12	**KRIVQRIKDFLR**-amide	Originally reported KR-12. Short length, narrow-spectrum activity, non-toxic. Need D-amino acids for stability	[32]
2013	KR-12R	**RRIVQRIRDFLR**-amide	Enhanced activity of an all-arginine analog	[43]
2013	KR-12-pa (KR-12-a3)	**KRIVKRIKKWLR**-amide	Enhanced activity. Need D-amino acids for stability	[44]
2016	Immobilized KR-12	C**KRIVQRIKDFLR**-amide	Immobilized KR-12 to prevent biofilm formation on the titanium surface. Longevity of implants is unknown	[45]
2016	KR-12/nanofiber	C**KRIVQRIKDFLR**-amide	Immobilized KR-12 inhibited biofilm formation, enhanced cell attachment, and conferred anti-inflammatory function (for wound healing)	[46]
2017	KR-12-a5	**KRIVKLILKWLR**-amide	Enhanced activity. Need D-amino acids for stability	[47]
2020	TAT-KR-12	YGRKKRRQRRR**KRIVQRIKDFLR**	Added cell penetration ability by increasing peptide length. Need D-amino acids for stability	[48]
2020	Lipidated KR-12	**C8-KRIVQRIKDFLR**-amide	Optimal activity when coupled with the C8 fatty acid	[49]
2021	Trp cage KR-12	**KRIVQRIKDFLR**KYAQWLADGGPSSGRPPPK	KR-12 with stability but longer peptide sequence	[50]
2021	C10-KR8d (smaller than KR-12)	C10-**KRIWQRIK**-amide	KR-8 lipopeptide with potency, selectivity, and stability (all D-amino acids)	[51]
2022	CD4-PP	CPGG**KRIVKRIKAFLR**GPGG**KRIVKRIKAFLR**	Head-to-tail cyclic dimeric KR-12. Stable, potent, less selective, increased cost to synthesize	[52]
2023	CPP-KR12@Si	RKKRRQRRRGSS**KRIVQRIKDFLR**	Self-trapped CPP-KR12 silica particles with enhanced activity, cell-penetrating ability, increased stability for controlled release. Increased cost of peptide synthesis and cytotoxicity	[53]
2024	Long-CBP-KR12	**KRIVQRIKDFLR**GSGSGGSCQVLNPWYSQTTPGWGQC	KR-12 with carbohydrate-binding property. Costly to synthesize	[54]
2024	Stapled KR-12	**KRIVQRIKDFLR**-amide (i, i+4 Q22-D26)	Stability, selectivity, potency. Can be chemically synthesized	[55]

^1^ In the table, the amino acid sequence of KR-12 (also KR-8) is bolded and mutated amino acids are underlined. A more complete list of KR-12 peptides (Table S1) is provided as Supplementary Material.

**Table 2:** In vitro and in vivo assessment of efficacy and toxicity of KR-12 and its derivatives.

No.	Peptide ^1^	MIC	In Vitro Cytotoxicity ^2^	Type of Study	In Vivo Model	Treatment Dose	Refs.
1	Myr-KR-12N	4–16 μg/mL	RBC: HC_50_ > 100 μg/mL	1. Systemic toxicity 2. *E. coli* induced sepsis model	Mice	240 μg/mouse	[103]
	Myr-KR-12C	8–32 μg/mL			Mice	10 μg/mouse	[103]
2	KR-12-a2	1–8 μM	RBC: HC_50_ > 800 μM; RAW264.7: LC_50_ > 100 µM	Topical Ototoxicity	Guinea pigs	10 μg/mouse	[80,139]
3	C10-KR8d	1.5–25 μM	RBC: HC_50_ > 300 μM; HaCaT: LC_50_~96 µM	1. Systemic toxicity	Mice	40 mg/kg	[51]
				2. *S. aureus* Sepsis model	Mice	5 mg/kg	
				3. Catheter-associated biofilm model	Mice	250 μg/mouse	
4	Hyaluronic acid, Tannic acid, and KR-12 -Cryogel	3.2 mg of Cryogel	RBC: HC_50_ > 3.2 mg Cryogel; HaCaT and NIH-3T3 non toxic at 3.2 mg Cryogel	Wound infection model	Mice	2 mg/mL	[140]
5	Silk-fibroin-KR-12	Formulation 20 mg	L929 fibroblasts: LC_50_~5 μg/mL	Wound infection model	Mice	2 mg/mL	[141]
6	LL-37mini	8–64 μM	RBC: HC_50_ > 200μM; HaCaT LC_50_~100 µM	Wound infection model	Mice	10 mg/kg per wound	[83]
7	Stapled KR-12 (Q_5_, D_9_)	8–128 μM	RBC: HC_50_ > 256 μM; NIH-3T3: LC_50_~168.5 µg/mL	Wound infection model	Mice	160 μg/mouse	[55]
8	KR-12-a5	1–8 μM	RBC: HC_50_ > 800 μM; L929 LC_50_ > 500 ug/mL; RAW264.7: LC_50_~12.5 µM	LPS-induced bone erosion model	Mice	2 mg/kg	[142,143]
9	PEEK-PDA-KR-12	Formulation with 1 mg/mL	RBMSCs~1 mg formulation	Osteointegration evaluation model	Rats	1 mg/rat	[144]
10	KR-12	2.5–10 μM	RBC: HC_50_ > 340 μM; HBMSCs LC_50_~1000 µM	Induced colitis model	Mice	5 mg/kg	[51,145,146]

^1^ Peptide sequences can be found in Table 1 and Table S1; ^2^ In vitro toxicity was evaluated with RBC and other cell lines such as HaCaT. Peptide concentrations lysed 50% RBC and other mammalian cells are represented as HC_50_ and LC_50_, respectively.

## Supplementary Materials

The following supporting information can be downloaded at: https://www.mdpi.com/article/10.3390/antibiotics13090816/s1, Table S1. Amino acid sequences of KR-12 and its derivatives. Figure S1. The NMR structure of *E. coli* LPS-bound KR-12 determined by transferred NOEs. Reference [167] is only cited in the Supplementary Materials.
