# Supplementary material for: Origami of KR-12 Designed Antimicrobial Peptides and Their Potential Applications
Source: Antibiotics (Basel). 2024 Aug 28;13(9):816. doi: 10.3390/antibiotics13090816 (PMC11429261; doi:10.3390/antibiotics13090816)

Supporting information for *Antibiotics*

Origami of KR-12 Designed Antimicrobial Peptides and Their Potential Applications

Jayaram Lakshmaiah Narayana, Abraham Fikru Mechesso, Imran Ibni Gani Rather, D. Zarena, Jinghui Luo, Jingwei Xie and Guangshun Wang*

Department of Pathology, Microbiology, and Immunology, College of Medicine, University of Nebraska Medical Center, 985900 Nebraska Medical Center, Omaha, NE 68198-5900, USA

Contents

1. Table S1. A list of KR-12 derived peptide sequences
2. Figure S1. Structure of KR-12 in the presence of a small amount of *E. coli* LPS

Table S1. Amino acid sequences of KR-12 and its derivatives

No.	Peptide	Amino Acid Sequence	Ref.
1	KR-12	KRIVQRIKDFLR-NH_2	[32]
2	KR-12A18	**A**RIVQRIKDFLR	[43]
3	KR-12A19	K**A**IVQRIKDFLR	
4	KR-12A23	KRIVQ**A**IKDFLR	
5	KR-12A25	KRIVQRI**A**DFLR	
6	KR-12A29	KRIVQRIKDFL**A**	
7	KR-12A18Q19	**AQ**IVQRIKDFLR	
8	KR-12K	K**K**IVQ**K**IKDFL**K**	
9	KR-12R	**R**RIVQRI**R**DFLR	
10	K1A	**A**RIVQRIKDFLR	[114]
11	R2A	K**A**IVQRIKDFLR	
12	I3A	KR**A**VQRIKDFLR	
13	V4A	KRI**A**QRIKDFLR	
14	Q5A	KRIV**A**RIKDFLR	
15	R6A	KRIVQ**A**IKDFLR	
16	I7A	KRIVQR**A**KDFLR	
17	K8A	KRIVQRI**A**DFLR	
18	D9A	KRIVQRIK**A**FLR	
19	F10A	KRIVQRIKD**A**LR	
20	L11A	KRIVQRIKDF**A**R	
21	R12A	KRIVQRIKDFL**A**	
22	R2K	K**K**IVQRIKDFLR	
23	I3K	KR**K**VQRIKDFLR	
24	V4K	KRI**K**QRIKDFLR	
25	Q5K	KRIV**K**RIKDFLR	
26	R6K	KRIVQ**K**IKDFLR	
27	I7K	KRIVQR**K**KDFLR	
28	D9K	KRIVQRIK**K**FLR	
29	F10K	KRIVQRIKD**K**LR	
30	L11K	KRIVQRIKDF**K**R	
31	R12K	KRIVQRIKDFL**K**	
32	Q5A,D9A	KRIV**A**RIK**A**FLR	
33	Q5K,D9K	KRIV**K**RIK**K**FLR	
34	Q5K,D9A	KRIV**K**RIK**A**FLR	
35	I3K,Q5K,D9K	KR**K**V**K**RIK**K**FLR	
36	Q5A,D9A,F10A	KRIV**A**RIK**AA**LR	
37	V4A,D9A	KRI**A**QRIK**A**FLR	
38	I3A,V4A,D9A	KR**AA**QRIK**A**FLR	

39	I7K,F10K,L11K	KRIVQRKKDKR	
40	I7K,L11K	KRIVQR**K**KDFR	
41	KR-12-a1	KRIVQRIKD**W**LR- NH_2	[80]
42	KR-12-a2	KRIVQRIK**KW**LR-NH_2	
43	KR-12-a3	KRIV**K**RIK**KW**LR-NH_2	
44	KR-12-a4	KRIV**KL**IK**KW**LR-NH_2	
45	KR-12-a5	KRIV**KL**I**LKW**LR-NH2	
46	KR-12-a6	**L**RIV**KL**I**LKW**LR-NH2	
47	KR-12-a7	KRI**RK**RIK**KW**LR-NH2	
48	KR-12-a8	KRI**RK**RIK**KWK**R-NH2	
49	KR-12-a5(5-^{D}K)	KRIV**K**LILKWLR	[47]
50	KR-12-a5(6-^{D}L)	KRIVK**L**ILKWLR	
51	KR-12-a5(7-^{D}L)	KRIVKL**L**LKWLR	
52	KR-12-pa	KRIV**K**RIK**KW**LR	[44]
53	KR-12–3	KRIV**KW**IK**K**FLR	[98]
54	[W$^{7]}$KR12-KAEK	KRIVQRWKDFLRKAEK	[99] [167]
55	Lf-KR (LfcinB6-KR12a4}	**RRWQWRP**KRIVKLIKKWLR-NH2	[101]
56	TAT-KR-12	YGRKKRRQRRR- KRIVQRIKDFLR	
57	TC-KKA	DKYAQWLADGGPSSGRPPPK	[50]
58	AMTC 31-6	**KRIVQRIKDFLR**KYAQWLADGGPSSGRPPPK	
59	Helix 19-6	**KRIVQRIKDFLR**KYAQWLA-NH2	
60	AMTC 26-4	RIKDFLRKYAQWLADGGPSSGRPPPK	
61	AMTC 26-6	GIVRIFKRLYKQWLKDGGPSSGRPPPK	
62	AMTC24-5	GIFKRLYKQWLKDGGPSSGRPPPK	
63	Ga-KR12	Ga-KRIVQRIKDFLR	[49]

64	Ac-KR12-NH2 (C2-KR12-NH2)	C_2- KRIVQRIKDFLR	[49]
65	C4-KR12-NH2	C4- KRIVQRIKDFLR	
66	C6-KR12-NH2	C_6- KRIVQRIKDFLR	
67	C8-KR12-NH2	C_8- KRIVQRIKDFLR	
68	C10-KR12-NH2	C_{10}- KRIVQRIKDFLR	
69	C12-KR12-NH2	C_{12}- KRIVQRIKDFLR	
70	C14-KR12-NH2	C_{14}- KRIVQRIKDFLR	
71	Benzoicacid-KR12-NH2	Benzoicacid-KRIVQRIKDFLR	
72	*trans*-Cinnamic acid-KR12-NH2	*trans*-Cinnamic acid-KRIVQRIKDFLR	
73	Myr-KR-12N	Myr-**GGG**KRIVQRIKDFLR-NH2	[103]
74	Myr-KR-12C	KRIVQRIKDFLR**GGGK**-Myr	
75	KR-12(R_2, R_6)	Ac-K**S**$_5$IVQ**S**$_5$IKDFLR-NH2	[76]
76	KR-12(I_3, I_7)	Ac-KR**S**$_5$VQR**S**$_5$KDFLR-NH2	
77	KR-12(Q_5, D_9)	Ac-KRIV**S**$_5$RIK**S**$_5$FLR-NH2	
78	KR-12(I_7, L_{11})	Ac-KRIVQR**S**$_5$KDF**S**$_5$R-NH2	
79	KR-12(K_8, R_{12})	Ac-KRIVQR**S**$_5$KDF**S**$_5$R-NH2	
80	KR-12(K_1, K_8)	Ac-**R**$_8$RIVQRI**S**$_5$DFLR-NH2	
81	KR-12(R_2, D_9)	Ac-K**R**$_8$IVQRIK**S**$_5$FLR-NH2	
82	KR-12(I_3, F_{10})	Ac-KR**R**$_8$VQRIKD**S**$_5$LR-NH2	
83	KR-12(V_4, L_{11})	Ac-KRI**R**$_8$QRIKDF**S**$_5$R-NH2	
84	KR-12(Q_5, R_{12})	Ac-KRIV**R**$_8$RIKDFL**S**$_5$-NH2	
85	retro-KR-12	RLFDKIRQVIRK	[94]
86	retro-cd4	AGGKRIVQRIKDFLRGAGGRLFDKIRQVIRKG	
87	retro-cd3	AG. KRIVQRIKDFLRGAG.RLFDKIRQVIRKG	

88	retro-cd2	A..KRIVQRIKDFLRGA..RLFDKIRQVIRKG	
89	cd4	AGGKRIVQRIKDFLRGAGGKRIVQRIKDFLRG	
90	cd3	AG.KRIVQRIKDFLRGAG.KRIVQRIKDFLRG	
91	cd2	A..KRIVQRIKDFLRGA..KRIVQRIKDFLRG	
92	cd4-(Q5K,D9K)	AGGKRIVKRIKKFLRGAGGKRIVKRIKKFLRG	
93	2retro-cd4	CGGRLFDKIRQVIRKGAGGRLFDKIRQVIRKG	
94	retro-ld4*	CGGKRIVQRIKDFLRGAGGRLFDKIRQVIRKG	
95	ld4*	CGGKRIVQRIKDFLRGAGGKRIVQRIKDFLRG	
96	retro-cd4*	CGGKRIVQRIKDFLRGAGGRLFDKIRQVIRKG	
97	cd4*	CGGKRIVQRIKDFLRGAGGKRIVQRIKDFLRG	
98	KR-12 (Q5K, D9A)	KRIVKRIKAFLR	
99	cd4-CCPP	CFLRGAGGKRIVCRIKAFLRGAGGKRIVKRIK	
100	cd4-CC	CFLRGPGGKRIVCRIKAFLRGPGGKRIVKRIK	
101	C14-KR10	KRIWQRIKDF	[51]
102	C14-KR8	KRIWQRIK	
103	C14-KR6	KRIWQR	
104	C14-KR4	KRIW	
105	C12-KR12	KRIWQRIKDFLR	
106	C12-KR10	KRIWQRIKDF	
107	C12-KR8	KRIWQRIK	
108	C12-KR6	KRIWQR	
109	C12-KR4	KRIW	
110	C10-KR12	KRIWQRIKDFLR	
111	C10-KR10	KRIWQRIKDF	
112	C10-KR8	KRIWQRIK	
113	C10-KR8d	KRIWQRIK	
114	C10-KR6	KRIWQR	
115	C10-KR4	KRIW	
116	C8-KR12	KRIWQRIKDFLR	
117	C8-KR10	KRIWQRIKDF	
118	C8-KR10d	KRIWQRIKDF	
119	C8-KR8	KRIWQRIK	
120	C8-KR6	KRIWQR	
121	C8-KR4	KRIW	
122	C6-KR12	KRIWQRIKDFLR	
123	C6-KR10	KRIWQRIKDF	
124	C6-KR8	KRIWQRIK	
125	C6-KR6	KRIWQR	
126	C6-KR4	KRIW	
127	LL-10	LLGDFFRKSK-NH_2	[83]
128	KE-10	KEKIGKEFKR-NH_2	
129	KR-8	KRIVQRIK-NH_2	
130	RK-9	RKSKEKIGK-NH_2	

131	RIK-10	RIKDFLRNLV-NH_2	
132	Hyaluronic acid, Tannic acid, and KR-12 -Cryogel	HA-TA-KRIVQRIKDFLR-NH_2	[140]
133	Silk-fibroin-KR-12	$RSF_{PF127--}$ KRIVQRIKDFLR-NH_2	[141]
134	PEEK-PDA-KR12	PEEK-PDA- KRIVQRIKDFLR-NH_2	[142]

*Myr represents myristic acid *Ga represents gallic acid * ^{D}-D amino acid substitution

Figure S1. NMR structure of KR-12 in the presence of a tiny amount of *E. coli* LPS (peptide:LPS molar ratio ~ 300:1) at pH 5.7 and 25°C assuming the M. Wt. of heterogenous LPS is 20 kDa. This structure was calculated based on 185 NOE cross peaks obtained from transferred NOESY (Tr-NOESY) at 100 ms. Data collection, processing, signal assignment, and structural calculations were done by following an establishing procedure for KR-12 structural studies in complex with D8PG (see ref. [32]). The amino acid numbering system as in its parent peptide LL-37 is used here for KR-12 (residues 18-29) labeled below in the structure. When residues 20-27 were superimposed, the backbone rmsd was 0.204 Å. In the presence of LPS, an amphipathic structure with multiple turns can be seen. This structure differs from the more regular helical structure of KR-12 bound to D8PG (Figure 3).

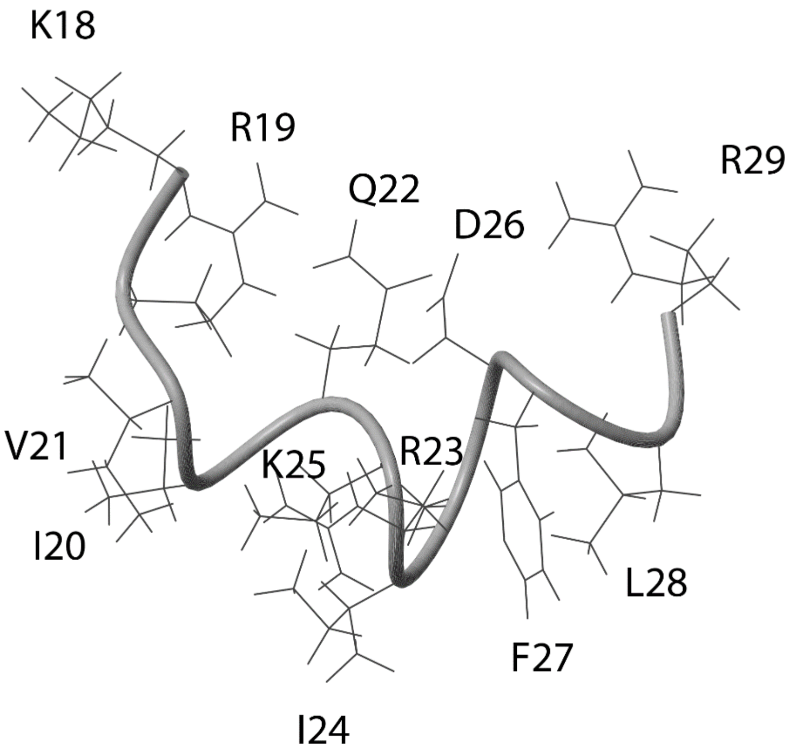

Supplement: Supplementary file 1 [file antibiotics-13-00816-s001.zip › antibiotics-3160974-supplementary.pdf]
